# Supplementary material for: Tight Interplay between Replication Stress and Competence Induction in Streptococcus pneumoniae
Source: Cells. 2021 Jul 30;10(8):1938. doi: 10.3390/cells10081938 (PMC8394987; doi:10.3390/cells10081938)
Supplement: Supplementary file 1 [file cells-10-01938-s001.zip › cells-1257978-supplementary.pdf]

Title

Tight interplay between replication stress and competence induction in *Streptococcus pneumoniae*

Vanessa KHEMICCI[1,2*] Marc PRUDHOMME[1,2*], and Patrice POLARD[1,2#]

[1] Laboratoire de Microbiologie et Génétique Moléculaires (LMGM), Centre de Biologie Integrative (CBI), Centre national de la Recherche Scientifique (CNRS), Toulouse France.

[2] Université de Toulouse, Université Paul Sabatier, Toulouse, France

[*] These authors have contributed equally to this work

[#]Corresponding author: patrice.polard@univ-tlse3.fr

Summary

Cells respond to genome damage by inducing restorative programmes, typified by the SOS response of *Escherichia coli*. *Streptococcus pneumoniae* (the pneumococcus), with no equivalent to the SOS system, induces the genetic program of competence in response to many types of stress, including genotoxic drugs. The pneumococcal competence regulon is controlled by the origin-proximal, auto-inducible *comCDE* operon. It was previously proposed that replication stress induces competence through continued initiation of replication in cells with arrested forks, thereby raising relative *comCDE* gene dosage and expression and accelerating onset of competence. We have further investigated competence induction by genome stress. We find that absence of RecA recombinase stimulates competence induction, in contrast to SOS, and that double-strand break repair (RexB) and gap repair (RecO, RecR) mediators do also, implying that recombinational repair removes competence induction signals. Failure of replication forks provoked by either titrating PolC polymerase with the base analogue HPUra, over-supplying DnaA initiator, or under-supplying DnaE polymerase or DnaC helicase stimulated competence induction. This induction was not correlated with concurrent changes in origin-proximal gene dosage. Our results point to arrested and unrepaired replication forks, rather than raised *comCDE* dosage, as a basic trigger of pneumococcal competence.

Keywords: DNA damage response; genome integrity; replication stress; recombinational repair; bacterial competence; *Streptococcus pneumoniae*.

Introduction

All genomes are susceptible to potentially life-threatening damage, of both environmental and endogenous origin. To confront this challenge to their survival, organisms have evolved a range of repair pathways, notably those inducible by the damage itself. The first program found to be triggered in response to DNA damage was the SOS response of *Escherichia coli* (Radman, 1975) (Witkin, 1976). It is set in motion when some mishap generates single-stranded DNA on which RecA protein polymerizes; the resulting nucleofilament activates autoproteolysis of the transcriptional repressor LexA and thus expression of the LexA-controlled genes that provide DNA repair functions.

The SOS system is widespread in bacteria but not universal, and some species have evolved other responses to genome damage (Erill et al., 2007). A prominent example is *Streptococcus pneumoniae* (the pneumococcus), which does not encode a LexA homologue but responds to genotoxic agents by developing the distinct physiological state of competence (Claverys et al., 2006). Competence provides the cells with new properties, including the well-known horizontal gene transfer process of genetic transformation (Dubnau and Blokesch, 2019). Many species share this ability to convert to competence for transformation, as well as a similarity of mechanism in the importing of external DNA across the cell membrane and integration into the genome by RecA-directed homologous recombination (Dubnau and Blokesch, 2019). On the other hand, regulation of competence appears specific to each species, reflecting the distinct integration of the genetic program into each bacterial lifestyle (Johnston et al., 2014b). Here, we report our analysis of pneumococcal competence

induction in response to various types of genome stress that disrupt DNA recombination and replication dynamics.

The core of pneumococcal competence control is a two-component system consisting of the histidine kinase ComD and the transcription regulator ComE (Johnston et al., 2014a)(Johnston et al., 2014b). The ComDE TCS is modulated by a competence-stimulating peptide (CSP), encoded in the form of a pre-CSP that is matured and exported through the cell membrane by the ABC transporter ComAB. These elements define a positive feedback loop, termed the ComABCDE core sensing system, which integrates various kinds of stress and coordinates development of competence in the population (Fig.1A;(Martin et al., 2013)). In liquid cultures, competence induction occurs only during the exponential phase of growth and after a time delay characteristic of the strain and growth conditions (a '*pre-competence*' period referred to as X_A; Fig. 1A (Prudhomme et al., 2016). Induction of competence results in two successive transcriptional waves, termed *early* and *late*. The early wave is initiated by ComE upon its phosphorylation by ComD (Martin et al., 2010)(Martin et al., 2013). Phosphorylated ComE activates about 20 early competence (*com*) genes, including *com*X whose product, the sigma factor σ^X, primes the late wave by governing transcription of about 60 further *com* genes. The σ^X regulon includes DprA that, in addition to mediating RecA-directed recombination during transformation (Mortier-Barrière et al., 2007), interacts with phosphorylated ComE to shut off competence (Mirouze et al., 2013). Thus, the shift to competence as measured by *com* gene expression is transient, lasting for roughly one generation (referred to as the X_B period (Prudhomme et al., 2016); Fig. 1A). A feature of pneumococcal competence is that it develops only during the exponential phase of growth, during which the replicative state of the chromosome renders it particularly sensitive to damage or disruption.

A previous study showed pneumococcal competence to be induced by drugs that alter genome integrity or replication (Prudhomme et al., 2006). Competence was proposed to substitute for the SOS system in the pneumococcus, which does not possess a LexA homologue (Claverys et al., 2006) (Charpentier et al., 2012). This raised the question of how replication stress signals are conveyed to the ComDE-mediated competence induction system. It was observed that these stresses were correlated with increased abundance of transcripts originating from the *ori*-proximal *com*CDE operon whose gene dosage had risen as a result of deceleration of the replication forks (Slager et al., 2014a). Here, we have further studied the signaling that triggers competence development following genome damage in the pneumococcus. We first found that RecA and the main homologous recombination pathways RexAB and RecFOR are not involved in this signaling. Rather, RecA prevents competence development *via* its recombinational repair activity. While we also observed a link between fork arrest and competence development, our results do not support the notion that signaling stems from a *com*CDE gene dosage effect. Instead, they suggest that arrested and unrepaired replication forks are the trigger for competence induction.

Results

RecA-mediated recombination prevents competence development in *S. pneumoniae*.

To compare competence development in wild-type (WT) and *rec*A mutant ($recA^-$) strains we monitored the expression of a competence-induced gene (*comC*) fused to the firefly luciferase gene, as reported previously (Prudhomme and Claverys, 2007); see M&M). In this assay, light emitted by the growing cells is recorded in real time as relative luminescence units (RLU) normalized to cell density expressed as OD_{492} (see

M&M; Fig. 1A). Under conditions appropriate for competence development, this gives rise to a characteristic, transient peak of competence gene expression during exponential growth. This expression, and competence development itself, declines progressively as the initial pH of the medium used is acidified (Fig S1; (Weyder et al., 2018)(Chen and Morrison, 1987)). Conditions that stimulate or depress competence induction, such as certain mutations and drugs, can overcome the pH effect and thus cause the return or disappearance of the expression peak, changes termed hereafter competence-up (‘*cup*’) and competence-down (‘*cdo*’) phenotypes, respectively. We observed that a *rec*A- mutant exhibits a *cup* phenotype, as it develops spontaneous competence at a pH at which the WT strain does not (Fig. 1B). This result implies that RecA protein impedes pneumococcal competence development.

As pneumococcal competence is known to be induced by various types of DNA damage (Prudhomme et al., 2006)(Slager et al., 2014a), the *cup* phenotype of the *recA* strain could stem from types of damage that are no longer efficiently corrected by RecA recombination activity. RecA-mediated maintenance of genome integrity is known to proceed by distinct recombination pathways, triggered by distinct types of damage and involving specific sets of effectors (Cox, 2007)(Kowalczykowski, 2015)(Michel and Leach, 2012). The early effectors of these pathways are recombination mediators that promote the formation of the RecA presynaptic recombination filaments on ssDNA, upon which RecA then executes the DNA strand-exchange reactions that restore the genome. Two distinct recombination mediators are involved in genome maintenance in bacteria. One is the RecF-RecO-RecR triad that acts at DNA gaps (Michel and Leach, 2012); the other is the RecBCD complex in *E. coli (Michel and Leach, 2012)*, termed RexAB in *S. pneumoniae* (Halpern et al., 2004), which acts at double-strand breaks (DSB). To evaluate the relative importance of these two recombinational

pathways in *S.pneumoniae*, we monitored competence induction in *recO*$^-$, *rec*R$^-$ and *rex*B$^-$ mutants by recording the expression of a *luc* transcriptional fusion with the *ssb*B competence gene. Each mutant exhibited a *cup* phenotype (Fig. 1B). These results show that impairment of recombinational pathways that repair the genome is a potential source of competence induction.

The *cup* phenotype of the *recA*$^-$ mutant is exacerbated by the DNA damaging agent Mytomycin-C in a dose response manner.

To further verify that the *cup* phenotype of the *rec*A$^-$ mutant results from a deficiency in repairing genome damage, we compared competence development of WT and *recA*$^-$ strains in the presence of increasing amounts of mitomycin-C (MMC), a DNA damaging agent known to induce pneumococcal competence when added at concentrations sub-inhibitory for growth (Prudhomme et al., 2006). Here, the pH of the medium was adjusted to prevent spontaneous competence development in *rec*A$^-$ cells. In these conditions competence of the WT strain was readily induced by MMC at 40 ng.ml^{-1} and above (Fig. 2). Competence was also induced by MMC in the *rec*A$^-$ strain, but at much lower concentrations of the drug (Fig. 2). Furthermore, the competence peak of the *rec*A$^-$ strain occurred earlier during growth than for the WT strain at all MMC concentrations tested, concomitant with an increase in induction level in proportion to MMC concentration. This result is likely to be a consequence of a gradual accumulation of unrepaired DNA lesions that foster competence development. In WT cells, most of these DNA lesions would be efficiently repaired by RecA recombination pathways, avoiding competence development until the extent of damage exceeds the capacity for efficient repair. Thus, these results support the notion that signaling

leading to competence induction is a quantitative response, developing in proportion to the damage suffered by the DNA.

Competence induction by replication inhibitor HpUra is independent of gene dosage changes.

Several drugs that inhibit replication have been reported to induce competence in Pneumococcus (Prudhomme et al., 2006)(Slager et al., 2014a). Chromosome replication in *S. pneumoniae* is initiated bidirectionally from the origin, *ori*C, situated between the two closely linked competence control operons, *com*CDE and *com*AB, and terminates diametrically opposite in the *ter*C region (Fig. S2). An analysis of transcription levels in cells treated with replication inhibitors suggested that the increased gene dosage of origin-proximal genes resulting from continued initiation on chromosomes with paused replication forks would raise the relative rate of *com*CDE gene expression enough to activate the ComABCDE positive feedback loop, leading to competence induction (Slager et al., 2014a). In this study, however, replication drugs were used at only one concentration, leaving open the possibility that other mechanisms might not have come to light.

We explored competence development over a range of concentrations of the replication damaging agent, HpUra, whose nucleotide derivative specifically blocks PolC polymerase progression by competing with dGTP (Brown, 1970). The addition of increasing amounts of HpUra to the WT strain gradually reduced the growth rate of WT cells (Fig. 3A). We monitored competence development over this range of HpUra concentrations in a medium adjusted to a pH that does not allow spontaneous competence development without the drug. Fig. 3A shows that *ssbB::luc* expression increased steadily with HpUra concentration up to 300 $ng.ml^{-1}$, at which point the

competence of the population was fully developed. We next determined the effect of HpUra concentration on relative *ori*C gene dosage, by qPCR measurement of the *ori*/*ter* ratio (see Fig. S2A and M&M). Fig. S2B shows the time course of *ori*/*ter* response to addition of HpUra at 50 ng.ml^{-1} in comparison with the *ori*/ter ratio of cells grown without the drug. In the absence of HpUra the *ori*/*ter* ratio remains constant at 1.6 (Fig. S2B), corresponding to the value obtained by sequencing of chromosomal DNA extracted from exponentially growing pneumococcal cells (Johnston et al., 2013). In the presence of HpUra, the *ori/ter* ratio gradually increased over time (Fig. S2B). Based on this calibration, we measured the *ori*/*ter* ratio over the whole range of HPUra concentration, in samples taken at the last time-point in the series (60 minutes). As shown in Fig. 3B, the correlation between *ori*/*ter* ratio and HpUra concentration breaks down above 100 ng.ml-1, whereas competence gene expression continues to rise. Indeed, at the highest HpUra concentration (1000 ng.ml^{-1}), the *ori/ter* ratio was nearly identical to that in the absence of drug (Fig. 3B) despite inducing the highest level of competence (Fig. 3A).

In the light of these results, we also measured the *ori/ter* ratio of WT cells grown in the presence of increasing amounts of MMC. In this case, the *ori/ter* ratio increased proportionally to the dose of MMC, with an inducible value of competence development above 40 ng.ml^{-1} (Fig. S2C).

These results show that an increase in the *ori/ter* ratio resulting from replication impediments, even massive, is not necessarily correlated with induction of competence. This implies that increased *com* gene dosage at *ori*C is not a sufficient, or perhaps even necessary, signal for competence development. Rather, competence gene expression is readily induced at levels of HpUra that provoke replication fork arrest, as seen by the sharply decreased *ori/ter* ratio and of growth rate at the highest

HpUra concentrations tested. These findings indicate that competence development is poised to respond to disruptions in DNA replication. We further explored this idea by examining the induction of competence in response to other essential replication effectors.

Modulation of competence development by altering DnaA concentration.

DnaA is the widely conserved master regulator of chromosomal DNA replication initiation in bacteria. Its activity is regulated by several mechanisms, some species-specific, which combine to coordinate replication with the cell-cycle. One key parameter is the concentration of DnaA molecules, alteration of which leads to replication stress through over- or under-initiation at *ori*C (Ogura et al., 2001).

To investigate whether artificial modulation of DnaA concentration in the cell could influence pneumococcal competence development, we replaced the promoter region of *dna*A with a synthetic IPTG-inducible P*lac* promoter (see M&M). Growth of the resulting ^{e}P*lac*-*dna*A strain was IPTG-dependent, as shown by the spot test assay presented in Fig. 4A. Cell growth was severely and progressively retarded at ≤ 2,5 µM IPTG, and scarcely any colony was detected with none. Above 5 µM IPTG, colonies were identical in form and number to those of the WT, but at the highest IPTG concentration tested (100 µM), colonies appeared smaller, indicating that *dna*A overexpression is detrimental to the cell cycle. The measured *ori*/*ter* ratios were correlated with colony-forming ability: *ori*C is under-replicated below 2.5 µM IPTG, is replicated at WT frequency at 5-10 µM and over-replicated at 100 µM (Fig. 4C). We then analyzed competence development in the ^{e}P*lac*-*dna*A strain grown in liquid medium with this range of IPTG concentrations and at different pH values. Cells grown

at the highest *dna*A expression level exhibited a *cup* phenotype in comparison with cells grown between 5 and 10 μM IPTG (Fig. 4B). In contrast, when grown in competence-permissive medium to allow a more graded observation of competence response, the ^{e}P*lac-dna*A strain developed competence later and less strongly at inducer concentrations below 2.5 µM than when induced at levels enabling normal growth (5-10 µM). We also noted that the X_A time taken to induce competence gradually decreases between 2.5 and 10 µM, while the *ori*/*ter* ratio is effectively constant (Fig. 4B). These results point to a correlation between *dna*A expression level and the tendency of cells to develop competence, independent of relative gene dosage.

The *dna*A gene is the first gene of an operon whose second gene is *dna*N, which encodes the DNA polymerase processivity factor of the replisome (Ogura et al., 2001). Consequently, DnaN concentration will also be modulated by IPTG concentration in the ^{e}P*lac-dnaA* strain, and this in turn could impact replication and the propensity of the cells to develop competence. Indeed, inhibition of DNA synthesis in *B. subtilis* by overproduction of DnaA was shown to result from DnaA-mediated repression of the *dna*A-*dna*N operon and the consequent depletion of DnaN protein (Ogura et al., 2001). Accordingly, we performed the same experiment in *S. pneumoniae* to explore how over-expression of DnaA alone affects competence development. We inserted an IPTG-inducible P*lac-dna*A construct at an ectopic position on the chromosome (see M&M). The resulting CEP-Pl*ac-dna*A strain was grown with 100 μM IPTG in a medium adjusted to a pH non-permissive for competence development without IPTG. As shown in Fig S3, DnaA over-expression readily induced competence, as well as hindering growth more severely than in the case of the ^{e}P*lac-dna*A strain at the same IPTG

concentration (see Fig. 4B). Thus, increasing the concentration of DnaA itself induces competence development.

We then investigated the *cdo* phenotype shown by the ^{e}Plac-*dna*A strain upon growth without IPTG (Fig. 4B). For this, we used a (*S. pneumoniae*) strain overproducing a close homologue of the *B. subtilis* YabA protein, which has been demonstrated to down-regulate replication initiation at *ori*C by interacting with DnaA (Noirot-Gros et al., 2006)(Merrikh and Grossman, 2011)(Soufo et al., 2008). Importantly, *B. subtilis* YabA has been shown not to interfere with transcriptional regulation by DnaA (Goranov et al., 2009). As shown in Fig. S4, we found that IPTG-induced expression of pneumococcal YabA from an ectopic P*lac-yab*A contruct led to a *cdo* phenotype. This result strongly supports the notion that a reduced rate of DnaA-mediated replication initiation at *ori*C depresses spontaneous competence development. This *cdo* phenotype could stem from diminished expression of the *com*CDE operon, resulting from reduction of the *ori*/*ter* ratio due to altered timing of replication from *ori*C. To explore this possibility, we determined whether ^{e}P*lac-dna*A cells grown without IPTG could develop competence upon addition of synthetic CSP to the growth medium. As shown in Fig. S5, CSP added at any time during growth led to immediate induction of competence in ^{e}P*lac-dna*A cells cultivated without IPTG or with 5 μM IPTG (in a medium of pH not permitting spontaneous competence development in the latter case). This indicates that DnaA-depleted cells maintained ComD and ComE basal levels above the threshold needed for CSP sensing and competence induction.

Taken together, these results support the idea of a cause-and-effect relationship between DnaA concentration and spontaneous development of pneumococcal

competence. They also indicate that the relationship is based on the rate of initiation from *ori*C rather than on regulatory properties of DnaA.

Aborted replicative DNA synthesis fosters competence development.

The experiments till this point revealed that alterations in the active concentrations of PolC and DnaA influenced spontaneous competence development. In both cases, a *cup* phenotype was observed under conditions leading to replication fork arrest and/or collapse. To delve further into this correlation, we used the same approach to examine the consequences of varying availability of the replicative helicase DnaC and the replisome polymerase DnaE, both being essential for cell viability. In *B. subtilis*, DnaC and DnaE intervene, in that order, prior to DnaN and PolC in assembly of replication forks at *ori*C and in re-initiation at interrupted forks, respectively (Sanders et al., 2010). Thus, depletion of either enzyme is expected to severely impede replication, at the initiation and the elongation steps, in both cases leading to accumulation of arrested forks. We constructed two strains to enable IPTG-dependent modulation of *dna*C and *dna*E expression at their native chromosomal loci. As expected, growth of these strains was IPTG-dependent (Fig. 5A and 6A). Notably, however, depletion of these proteins was not complete, as inferred from the increase in cell density of cultures even without IPTG (Fig. 5B and 6B). Growth under these conditions below 1μM IPTG is however not sufficient to generate visible colonies in solid medium (Fig. 5A and 6A). To measure spontaneous competence development in these strains over a range of IPTG concentration, we used cells initially cultured at an IPTG concentration permitting WT growth rate and *ori/ter* ratio and at a pH preventing induction of competence. Under these conditions, both the ^{e}P*lac*-*dna*E and ^{e}P*lac*-*dna*C

strains exhibited a *cup* phenotype when grown below 1 μM IPTG (Fig. 5B and 6B). The *ori*/*ter* ratio increased slightly in the [e]P*lac*-*dna*E strain under these conditions (Fig. 5C). In stark contrast, the *ori*/*ter* ratio fell markedly in the [e]P*lac*-*dna*C strain grown below 1 μM IPTG (Fig. 6C), *i.e*. in the conditions leading to competence development.

Taken together, these results reinforce the notion that replication fork arrest and collapse are major triggers of competence development in *S. pneumoniae*.

Discussion

In this study, we have investigated the causal relationship between damage to the integrity or replication of the genome and competence induction in *S. pneumoniae*. We observed that deficiencies in RecA-directed repair stimulated competence development, and conclude that competence induction stems from the sensing of injuries that are continuously generated during growth but normally repaired. Recombinational DNA repair pathways are known to buttress chromosomal DNA replication by promoting the rescue of damaged forks, *via* mechanisms that differ according to the type of injury suffered (Michel and Leach, 2012)(Michel et al., 2018). Thus, the *cup* phenotype of the *rec*A, *rec*O, *rec*R and *rex*A mutants would reflect their failure to efficiently repair damage at replication forks (Fig. 1B). Supporting this proposal, a *cdo* phenotype appears upon inhibition of DnaA-mediated initiation (by overexpression of the YabA replication repressor; Fig. S4): the lower initiation rate would reduce the frequency of forks, and hence of fork failure and the signals triggering competence. Failure of repair was not the only source of damage signals: increasing damage frequency by addition of replication inhibitors, such as MMC and HpUra, also accelerated competence development. Common to all the disruptions of replication that lead to competence development are unrepaired replication forks.

How does replication stress induce competence?

Previous studies that linked replication stress to pneumococcal competence induction used exogenous drugs to specifically alter either a replication protein, the dNTP pool, or DNA integrity (Prudhomme et al., 2006)(Slager et al., 2014b). Among these drugs, the most specific for the replication process is HpUra, which effectively reduces the concentration of active replisomal PolC DNA polymerase in a dose response manner. Here, we constructed strains that express the DnaC helicase and the DnaE DNA polymerase of the replisome in proportion to the concentration of added IPTG. The diminution of the active pool of DnaC and of DnaE molecules led to the induction of competence, mirroring the effect of high concentrations of HpUra on WT cells (Fig. 5, 6 and 3).

It was previously proposed that the replication defect caused by HpUra was converted into a competence inducing signal as a consequence of the higher relative dosage of the *ori*-proximal *com*CDE operon resulting from deceleration of the replication forks. HpUra-treated cells were shown to express the *com*CDE operon at higher levels than untreated cells, and this was proposed to activate the ComABCDE positive feedback loop and turn on competence (Slager et al., 2014a). A central parameter of this mechanism is the *ori*/*ter* ratio, which should increase under replication stress and be correlated with competence induction. We found instances in which this correlation does not hold. An extreme case was that of the ^{e}P*lac*-*dna*C strain, which exhibited a *cup* phenotype when grown with concentrations of IPTG at which the *ori*/*ter* ratio gradually declined (Fig. 6). Indeed, the *ori*/*ter* ratio varied for each replication stress and was not correlated with the propensity of the cells to develop

competence. Thus, a high *ori*/*ter* ratio, > 3, was found with HpUra at 50 $ng.ml^{-1}$, which however was not followed by competence induction (Fig. 3). By contrast, almost no change in the *ori*/*ter* ratio was found for WT cells grown with 1000 $ng.ml^{-1}$ HpUra (Fig. 3) or for the ^{e}P*lac*-*dna*E cells grown without IPTG (Fig. 5), although competence was readily induced in both situations. Thus, rather than raised *com*CDE gene dosage and expression, the factor common to all situations of replication stress that foster competence induction was the arrest of replication forks.

In several cases, the propensity of the cells to develop competence was found to be proportional to the intensity of stress applied. The gradualness of the response was manifested by a change in timing of competence induction, *i.e.* the more stress, the shorter the period (X_A) preceding the peak of expression of competence genes. This was observed in experiments with increasing concentrations of MMC applied to *recA*$^{-}$ cells (Fig. 2), and to ^{e}P*lac*-*dna*E and ^{e}P*lac*-*dna*C cells grown with 1 μM and 0 μM IPTG (Fig. 5 and 6). Importantly, this dose-response effect should be considered in the light of the two-phase development of competence in pneumococcal populations and the mechanism underlying its coordination between cells. We previously provided genetic evidence that competence of a Pneumococcus population develops in a self-activating (autocrine) stage, followed by a wholesale propagation (paracrine) stage (Fig. 1A;(Prudhomme et al., 2016). During the initial phase, there is a steady increase in the number of individual cells that undergo physiological stress of a kind that induces the ComABCDE feedback loop. When the fraction of such cells rises to a certain level, CSP-mediated induction of neighboring cells through cell-to-cell contact becomes frequent enough to thrust the population into the second phase where competence spreads throughout, as seen by a peak upon monitoring of competence gene expression. The proportional response of competence induction to replication stress

reported here is readily explained in this scenario. Lowering IPTG concentrations in cultures of ^{e}P*lac-dna*E and ^{e}P*lac-dna*C strains would be expected to progressively reduce DnaE and DnaC levels and increase the frequency of fork stalling. The fraction of individual cells that switch to competence would thus rise faster, leading to the shorter X_A periods observed. Similarly, addition of increasing amounts of MMC to *recA*$^{-}$ cells will raise the fraction of cells accumulating unrepaired damaged DNA, thus progressively reducing the period of competence development (Fig. 2). Notably, this gradual response is not observed for WT cells treated with the same range of MMC concentration (Fig. 2). In this case, recombinational repair is fully active up to a limit of MMC concentration above which competence is induced, reflecting a saturation of repair capacity and leading to a maximal X_A value. The same reasoning applies to ^{e}P*lac-dna*A cells grown at low IPTG concentrations. A gradual reduction of the replication initiation rate at *ori*C resulted in an extended X_A period of spontaneous competence development (Fig. 4). The same effect was obtained with WT cells by overexpression of the replication inhibitor YabA (Fig. S4). In both cases, the gradual diminution of the fraction of cells undergoing active replication exerts a corresponding delay in competence development.

Comparison of pneumococcal competence with the SOS response.

Unlike many bacteria, the pneumococcus does not have an SOS system to respond to DNA damage. Competence, like the SOS response, involves the induction a large set of genes that confer altered properties on the cell, but except for a small subset of proteins for DNA repair and recombination, there is little similarity in the competence and SOS regulons. Indeed, *rec*A is the only gene induced in both systems.

Nevertheless, our study of competence development in response to DNA damage reinforces the notion that competence is the functional equivalent of SOS. Both systems induce a cell division inhibitor that provides a delay to enable cells to repair DNA breaks before division. In the case of the pneumococcus, the ComM division inhibitor induced during competence was shown to be crucial for maintaining genome integrity during natural transformation (Bergé et al., 2017). Competence for transformation itself has been reported to improve survival to exposure to MMC (Engelmoer and Rozen, 2011), underlining the functional parallel of cell rescue between competence and SOS.

Concluding remarks

This study focuses on the relationship between induction of competence in the pneumococcus by replication stress. The underlying signaling mechanism appears not to depend on an increase in the copy number of the *ori*C-proximal *com*CDE operon following replication slowdown, as proposed previously (Slager et al., 2014b). This leaves open the question of the molecular mechanism that links genome damage to the induction of pneumococcal competence. Aborted replication forks appear to be essential, and presumably trigger the signaling pathway that ultimately induces the ComABCDE positive feedback loop. Competence was shown also to be induced in response to genome damage in two other naturally transformable species, *Legionella pneumophila* and *Helicobacter pylori*, neither of them encoding a LexA homologue or possessing an SOS system (Charpentier et al., 2012). The regulatory circuit controlling competence in these species is poorly defined, but distinct from the pneumococcal one. Thus, it will be interesting to establish whether a common mechanism leads to

competence development in response to genome stress in these species, whatever the particulars of the pathway controlling competence gene expression.

Figures Legends

Figure 1: Pneumococcal competence development cycle: effect of repair-deficiency mutations.

A: Model of competence development in *S. pneumoniae*. The plot shows the development of competence in an exponentially growing culture (the growth curve is omitted for clarity). The core sensing and regulatory machinery of competence is composed of the *comA*, *comB, comC* (CSP), *comD and comE* gene products, which define a positive feedback loop referred to as ComABCDE. The green arrow and the black T-bar represent external positive and negative inputs, which balance the idling of the core sensing machinery. At some point, growth conditions will provide enough positive input to activate the ComABCDE positive feedback loopin an autocrine mode in some of the cells. Next, this fraction of cells propagates competence within the population in a paracrine mode by transmitting the CSP *via* cell-to-cell contact (Chen and Morrison, 1987). In this model, the autocrine and paracrine modes of competence development proceed in two distinct periods defined as X_A and X_B, respectively (Prudhomme et al., 2016). X_B is followed by shut-off of competence gene expression. The dashed grey lines represent competence gene expression during the X_A and X_B periods. **B**: RecA recombination repair pathways prevent competence development. Cells were grown in C+Y medium with initial pH adjusted with HCl to inhibit competence development of the reference strains R825 or R895 (blue curves); see also Fig. S1. Competence of the population is shown as solid lines ($RLU.OD_{492}^{-1}.10^3$)

and cell density as dashed lines (OD_{492}). Each panel shows a comparison of competence development of the WT strain with that of the isogenic *recA*$^-$ (red) R3309, *rexB*$^-$ (light brown) R4036, *recO*$^-$ (dark green) R2411 and *recR*$^-$ (orange) R2412 mutant strains. Data are the means of at least 5 independent experiments, with standard deviations shown for the RLU.OD_{492}^{-1} measurements.

Figure 2: RecA suppression of competence induction by Mitomycin C

WT and *rec*A$^-$ cells were cultivated in C+Y medium adjusted to a pH non-permissive for spontaneous competence development. Cell density (OD_{492}) is shown in the upper panels and competence gene expression (RLU.OD_{492}^{-1}.10^3) in the lower panels. After 70 minutes of growth, MMC was added at the indicated concentrations, shown as light grey-blue to dark grey-blue for WT cells and light brown to dark brown for *recA*$^-$ cells. The curves represent the mean of two replicates. Standard deviations are omitted for clarity. Double arrowheads depict the X_A period.

Figure 3: HpUra induces competence independently of its effect on the *ori/ter* ratio.

A: R895 cells were grown at a pH non-permissive for spontaneous development of competence. After 70 min of growth, HpUra was added at 0, 50, 100, 300, 600 and 1000 µM, light grey-blue to dark grey-blue, respectively. Cell density and competence plots are as in Fig. 2. Curves represent the mean of three replicates for each condition. Standard deviations are omitted for clarity. **B**: *ori*/*ter* ratios measured by qPCR on total DNA extracted from the WT R825 strain 1 hour after HpUra addition at 0, 10, 25, 50,

100, 300, 600 and 1000 µM (shown as light green to dark green respectively; see also Fig. S1). The *ori*/*ter* ratio obtained for cells grown without HpUra was set to 1 and used as the reference for *ori*/*ter* ratios of cells at each HpUra concentration. The competence development status of each assay is indicated by the blue (induced) and red (basal level) double arrowheads. The means and standard deviations were derived from two replicates.

Figure 4: Differential control of competence development by modulated *dnaA* expression.

In strain R3317, the endogenous *dnaA* promoter has been replaced by the inducible P_{lac} promoter ($^{e}P_{lac}$::*dnaA*, see M&M). **A**: Growth assay. R3317 cells were grown to OD550 = 0.3 and were serially diluted and spotted on plates containing the indicated IPTG concentrations. The bracket covers IPTG concentrations enabling the mutant to mimic *wt* growth. **B**: *ori*/*ter* ratios measured by qPCR on total DNA extracts from the R3317 strain ($^{e}P_{lac}$::*dnaA*) cultivated at the indicated IPTG concentrations and normalized to the *ori*/*ter* ratio of cells grown with 10 µM IPTG. The latter ratio also corresponds to the *ori*/*ter* ratio of the isogenic WT strain (red horizontal dotted line). The competence development status of each assay is indicated by the blue (induced) and red (repressed) double arrowheads (see M&M). The means and standard deviations were based on two replicates. **C**: Growth (upper graphs) and competence gene expression (lower graphs) of the wt and $^{e}P_{lac}$::*dnaA* strains (with 5 µM IPTG) were monitored in media non-permissive (left panels) or permissive (right panels) for competence development. Double arrowheads specify the X_A period. Curves represent the mean of two replicates. Standard deviations are omitted for clarity.

Figure 5: Reduction of *dnaE* expression induces competence.

In strain R3315 the endogenous *dnaE* promoter has been replaced by the inducible P_{lac} promoter ($^{e}P_{lac}$::*dnaE*, see M&M). **A**: Growth assay. R3315 cells were grown to OD550 = 0.3 then diluted and spotted on medium containing the indicated IPTG concentrations. The bracket covers IPTG concentrations enabling the mutant to mimic *wt* growth. **B**: *ori*/*ter* ratios measured by qPCR on total DNA extracted from the R3315 strain ($^{e}P_{lac}$::*dnaE*) cultivated at the indicated IPTG concentrations. The *ori*/*ter* ratio obtained for cells grown with 10 µM IPTG was used as the reference ratio to calculate Relative level (y-axis); it corresponds also to the *ori*/*ter* ratio of the isogenic WT strain (red horizontal dotted line). The relative *ori*/*ter* ratio at each IPTG concentration is indicated, with standard deviations: values are the means of two replicates. The competence development status of each assay is indicated by the blue (induced) and red (repressed) double arrows. **C**: Competence expression of the *ssbB::luc* transcriptional fusion in R3315 was monitored in C+Y medium supplemented with 10 µM IPTG, which fully complements growth, and adjusted at an initial pH non-permissive for competence development. Curves represent the mean of four replicates for each condition. Standard deviations are omitted for clarity. Double arrowheads specify the X_A period.

Figure 6: Reduction of *dnaC* expression induces competence and diminishes the *ori/ter* ratio.

In strain R3316 the endogenous *dnaC* promoter has been replaced by the inducible P_{lac} promoter ($^{e}P_{lac}$::*dnaC*, see M&M). **A**: Growth assay. R3316 cells were serially diluted and spotted on medium containing the indicated IPTG concentrations. The bracket covers IPTG concentrations enabling the mutant to mimic *wt* growth. **B**: *ori/ter* ratios measured by qPCR on total DNA extracted from the R3316 strain ($^{e}P_{lac}$::*dnaC*) cultivated at the indicated IPTG concentrations. The *ori/ter* ratio obtained for cells grown with 10 µM IPTG was arbitrarily set to 1 and used as reference ratio (relative level), which corresponds also to the *ori/ter* ratio of the isogenic WT strain (red horizontal dotted line). The relative *ori/ter* ratio measured in each condition is indicated. The competence development status of each assay is indicated by the blue (induced) and red (repressed) double arrows lines. The mean and standard deviation were generated on 2 replicates. **C**: Expression of the *ssbB*::*luc* transcriptional fusion in the WT strain R895 (blue) and its isogenic $^{e}P_{lac}$::*dnaC* strain R3316 (orange). Cell density (OD_{492}) is presented in dotted lines and competence ($RLU.OD_{492}^{-1}.10^{3}$) in plain lines. The strains were cultivated at 37°C in C+Y medium (with 10 µM IPTG for R3316) at a pH non-permissive for spontaneous development of competence. Double arrowheads specify the X_A period.

Figure S1: Monitoring pneumococcal competence development.

pH of the medium modulates pneumococcal competence development. The CSP-ComD-ComE module of the ComABCDE positive feedback loop is sensitive to pH

changes. Alkaline and acidic pH activates and represses competence induction, respectively. A range of the initial pH values ranging from 7.6 to 6.8 of the C+Y medium was set up by increasing the molarity of HCl (7, 8 ,9, 10, 11, 12, 13, 14 and 16 mM respectively dark blue to light blue) and competence was monitored with the WT strain R825. The top panel shows culture growth as OD_{492} and reveals that growth rate is unaffected by pH within the range tested. The middle panel shows kinetics of competence induction as a function of pH: the more acidic media (14 and 16 mM HCl) do not permit competence shift. The bottom panel shows the X_A time until competence induction for each assay with the same color gradation. NC: no competence development. Experiments shown are representative of several, all giving similar results.

Figure S2: Determination of the *ori/ter* ratio.

A: Schematic representation of the pneumococcal chromosome mid-way through replication. Coordinates are those of strain R6, reference NC_003098.1. The competence core sensing genes are highlighted in red. The primers (green arrows) used to estimate *ori*/*ter* ratios by quantitative PCR are shown with the coordinates of their 5' nucleotides. **B**: Cell density at 37°C (OD_{492}) are reported as described in M&M with the wt strain (R895). The strains were cultivated at 37°C in C+Y medium at a pH non-permissive for spontaneous development of competence. After 65 minutes of growth, HpUra was added at 0 or 50 µM (respectively light grey-blue and dark grey-blue curves). Curves represent the mean of three replicates for each condition. *ori*/*ter* black arrows show time of sampling for *ori*/*ter* determination. *ori*/*ter* ratios measured by qPCR on total DNA extracted from R825 wild-type strain at different times after

HpUra addition is represented in the right panel. The *ori*/*ter* ratio obtained for cells grown without HpUra is 1.6 and was used to normalize *ori*/*ter* ratios from HpUra-treated cells. Means and standard deviations were derived from 2 or 3 replicates of distinct culture samples. **C**: *ori*/*ter* ratios increase with exposure to MMC. *ori*/*ter* ratios measured by qPCR on total DNA extracted from R825 cells grown for 1 hour after addition of MMC at the indicated concentrations. The *ori*/*ter* ratios obtained were normalized as in panel B. The competence development status of each assay is indicated by the blue (induced) and red (repressed) double arrowheads.

Figure S3: *dnaA* overexpression slows growth and induces competence.

Expression of the *ssbB*::*luc* transcriptional fusion in the WT strain R895 (blue) and its isogenic derivatives R3318 (orange), which contains an additional copy of the *dnaA* gene under the P_{lac} promotor at the CEP insertion platform (giving the CEP-P_{lac}::*dnaA* construct; see M&M). Cell density at 37°C (dotted lines, OD_{492}) and competence (full lines, RLU.OD_{492}^{-1}.10^{3}) are reported as described in M&M. The strains were cultivated at 37°C in C+Y media at a pH non-permissive for spontaneous development of competence, without IPTG (left panel) or with 100 µM IPTG (right panel). Mean and standard deviation were calculated from five independent assays. Double arrowheads specify the X_A period.

Figure S4: *yabA* overexpression represses competence induction.

A: Competence development of R3319 strain containing an ectopic additional copy of the *yabA* gene under the P_{lac} promotor (CEP-P_{lac}::*yabA* construct; see M&M) without

addition of IPTG (blue) or with 100µM IPTG (orange). Left axes present competence (RLU.$OD_{492}^{-1}.10^3$) and right axes the cell density (OD_{492}) at 37°C as described in M&M. Cultures were grown in C+Y medium with addition of a range of HCl to modulate competence development (7, 8 ,9 and10 mM HCl). Curves represent the mean and standard deviation of two replicates. Double arrowheads specify the X_A period. **B**: the histogram represents the mean of the X_A in minutes for each experiment. NC: no competence development. The mean and standard deviation were generated from four independent replicates.

Figure S5: Response to CSP addition during reduction in *dnaA* expression.

R3317 ($^{e}P_{lac}$::*dnaA*) cells were first grown with 10 µM IPTG to OD_{492} 0.2 in C+Y medium containing 15 mM HCl to repress spontaneous competence induction. These cells were diluted 1000-fold into C+Y with15 mM HCl, without IPTG (orange), or with 5 µM IPTG (blue) that enables growth equivalent to the WT isogenic strain (see Fig. 4). CSP was added (100 ng.ml^{-1}) to parallel diluted cell cultures at the times indicated (black arrows). Growth (dashed lines) and competence (full lines) were monitored in the resulting cultures. The curves represent the mean of 3 experiments, which gave similar results (standard deviation is shown only for competence as a matter of clarity).

Material and methods

Pneumococcal strains, cell growth, competence recording and transformation procedures.

Streptococcus pneumoniae strains used in this study are listed in Table S1. They are all R800 derivatives, which derivates from the unencapsulated R6 strain originating from D39 lineage (Prudhomme et al., 2016).

All strains were stored at -70°C in the form of stock cultures, which were used to inoculate liquid fresh medium to measure competence development under the distinct conditions described in the text and/or the figures legends. Stock cultures were prepared at 37°C in liquid C+Y medium at pH 6.8 to impede spontaneous competence development. Cells were grown to OD$_{550}$ = 0.2, centrifuged at 6000 rpm during 5 minutes at 4 °C to discard the medium, resuspended with fresh C+Y medium containing 15% glycerol to OD$_{550}$ = 0.4 and kept frozen at −70°C. In the case of $^{e}P_{lac}$::*dnaA*, $^{e}P_{lac}$::*dnaE,* and $^{e}P_{lac}$::*dnaC* strains, C+Y medium was supplemented with 10 µM IPTG, which is the concentration that results in a WT growth rate *and ori*/*ter* ratio value.

Competence was monitored in real time over the growth of the cell population by using a transcriptional fusion between the *luc* firefly luciferase gene and the *com*C promoter (in the case of the *rec*A$^-$ and *rex*B$^-$ strains, along with the isogenic WT strain) or *ssb*B promoter (in all other mutant strains, along with the isogenic WT strain), as listed in Table S1. Cultures were started by diluting the stock cultures 50 or 100 folds in C+Y medium adjusted to the desired pH and supplemented with luciferin in a 96-well white NBS micro plate (Corning) (Prudhomme and Claverys, 2007). Relative

luminescence units (RLU) and OD_{492} values were recorded at 37°C in a Varioskan Flash luminometer (Thermo 399 Electron Corporation).

HpUra, MMC and IPTG were added to the growth medium at the final concentrations indicated in the figures. OD_{492} values and luminescence values reported as RLU/ OD_{492} ratio have been measured in real time over the growth of each strain in the different conditions described in the figures. Each figure reports a representative experiment or a mean (and standard deviation) of at least three independent experiments, which gave rise to similar results.

For the plating tests, stock cultures were 10-fold serially diluted, spotted on CAT-agar containing 2% horse blood, 500 units/ml of catalase and IPTG (as indicated) and incubated overnight at 37°C.

Genome modifications were performed by natural transformation with the use of chromosomal or PCR fragments as transforming DNA (tDNA), following the procedure described previously (Martin *et al.*, 2000). Briefly, freshly inoculated stock cells were treated at 37°C for 10 min with synthetic Competence-Stimulating Peptide (CSP1; 100 ng ml^{-1}) to induce competence for genetic transformation. Next, tDNA was added to the competent cells, which were further incubated for 20 min at 30°C before plating on CAT-agar plates supplemented with 4% horse blood and, when required, with the appropriate concentration of the following antibiotics and after phenotypic expression for 120 minutes at 37°C: kanamycin (Kn; 500 µg ml^{-1}), spectinomycin (Spc; 200 µg ml^{-1}), chloramphenicol (Cm; 9 µg ml^{-1}). Constructs obtained with PCR fragments as tDNA were checked by PCR and sequencing.

Pneumococcal strains constructions.

*rec*A, *rec*O, *rec*R, *rec*F, *rex*B strains.

The *recA*$^-$ R3309 strain was constructed by transformation of the R825 strain with genomic DNA of R209 strain that contains the *recA*::*cm* allele. The *recO*$^-$ strain R2411 was constructed by transformation of the R895 strain with genomic DNA of R2372 strain that contains the *recO*:: *spc*^{13C} allele (Johnston *et al.* 2015). The *recR*$^-$ strain R2412 was constructed by transformation of the R895 strain with genomic DNA of R2373 strain that contains the *recR*:: *kan*^{15C} allele (Johnston *et al.* 2015).The *rexB*$^-$ strain R4036 was constructed by transformation of the R825 strain with genomic DNA of DPH14 strain that contains the *rexB*:: *spc* allele (Halpern *et al.* 2004).

<u>CEP-P*lac*::*yab*A strains.</u>

The CEP-P_{lac}::*yab*A strain (R3319) derives from the R895 and R3310 strains. The R3310 strain comprises the Chromosomal Expression Platform (CEP)-P_{lac}, which is inserted at the *amiF* locus and is composed of a Kn-resistance gene, the *lacI* gene, the IPTG-inducible P_{lac} promoter and the *luc* coding sequence as previously described in Johnston *et al*. 2018. *The* CEP-P*lac*::*yab*A (R3319) strain was generated by transforming the R895 strain with a SOEing PCR product composed of the *CEP-*P_{lac}*::yabA* sequence framed by the *ami*F-*lac*I-P_{lac} and *kan-tre*P sequences and obtained from the three PCR products : *ami*F-*lac*I-P_{lac} (primers OVK134 and OVK169 using R3310 genomic DNA as a template), *yab*A (primers OVK229 and OVK230 using R895 genomic DNA as a template) and *kan-tre*P (primers OVK172 and OVK139 using R3310 genomic DNA as a template).

<u>CEP-P*lac*::*dna*A strain</u>

The CEP-P_{lac}::*dna*A strain (R3318) was obtained by transformation of the R895 strain with plasmid pVK$^{\text{CEP-P}lac::dnaA}$, which includes the *lac*I-P_{lac}*::dna*A-*kan* construct framed by *ami*F and *tre*P sequences constructed by the SOEing with the pUC19

plasmid backbone . This plasmid derives from the plasmid pVK$^{CEP\text{-}Plac::I\text{-}sceI}$ constructed as follow: first, a CEP-P_{lac}::I-*sce*I strain (R3312) has been constructed by transformation of R825 with *a* SOEing -PCR fragment assembled from three PCR products corresponding to *ami*F-*lac*I-P_{lac} *(*primers OVK134 and OVK169 and genomic DNA from R3310 as template), I-sceI (primers OVK170 and OVK208 and plasmid pUC19-I-SceI as template) and *kan-tre*P (primers OVK172 and OVK139 using genomic DNA of R3310 as template (the I-*sce*I coding sequence was synthesized with codons optimized for *S. pneumoniae* as defined by the OptimumGene™ algorithm and cloned into pUC19 vector by Genscript USA to generate plasmid pUC19-I-*sce*I); second, a PCR fragment corresponding to CEP-P_{lac}::I-*sce*I (primers OVK301 and OVK304 using genomic DNA from R3313 strain) digested PstI/EcoRI was cloned into pUC19$^{(NdeI)}$ (pUC19$^{(NdeI)}$ is a pUC19 derivative mutated for its NdeI site); third, to generate the pVK$^{CEP\text{-}Plac::dnaA}$ plasmid, a PCR fragment encompassing *dna*A coding sequence, generated with OVK214 and OVK3 primers and using R895 as template, has beed digested by NdeI and BamHI and substituted to I-SceI coding sequence into pVK$^{CEP\text{-}Plac::I\text{-}sceI}$ digested by NdeI and BamHI.

$^eP_{lac}$::dnaA, $^eP_{lac}$::dnaE, $^eP_{lac}$::dnaC strains

To construct these strains, we first designed a construct allowing the targeted insertion of the P_{lac} promoter at any endogenous locus of the pneumococcal chromosome and defined as $^eP_{lac}$ hereafter (with ^{e} standing for endogenous). This construct derived from the CEP-P_{lac}::*luc* ectopic construct of the R3110 strain (Johnston et al. 2018), with a Spc-resistance gene (*aad9*) substituting the Kn-resistance gene to give the *R3311* strain. This substitution was achieved by transformation of the R3110 strain with a SOEing-PCR product between three PCR products corresponding to *lac*I (primers OVK136 and OVK213 using genomic DNA of R3310 as template), *aad*9 (primers

OVK210 and OVK28 on plasmid pR412 (Johnston et al., 2016), and P_{lac}::*luc* (primers OVK214 and OVK215 using genomic DNA of R3310 as template), and selecting for Spc^{R} Kn^{S} clones.

The $^{e}P_{lac}$::*dna*A strain (R3317) was obtained by transformation of R895 with a SOEing PCR fragment generated from three overlapping PCR products made of the 5'-*dnaA* sequence (primers OVK196 and OVK218 using genomic DNA of R895 as a template), the *lac*I and P_{lac} promoter sequences up to the ATG initiation codon (primers OVK136 and OVK169 using genomic DNA of R3311 as template) and the *dna*A coding sequence (primers OVK219 and OVK199 using genomic DNA of R895 as template), and selection for *Spc*^{R} clones. Of note, the natural TTG start codon of *dna*A has been maintained in this synthetic construct and is preceded by the ATG codon brought by the P_{lac} promoter.

The $^{e}P_{lac}$::*dnaC* (R3316) strain was obtained by transformation of R895 with a SOEing PCR product generated from three overlapping PCR products made of the *5'-dnaC sequence* (primers OVK245 and OVK246 using genomic DNA of R895 as template), the *lac*I and P_{lac} promoter sequences up to the ATG initiation codon (primers OVK136 and OVK237 using genomic DNA of R3311 as template) and the *dna*C coding sequence (primers OVK247 and OVK248 using genomic DNA of R895 as template).

The $^{e}P_{lac}$::*dnaE* (R3315) strain was constructed by transformation of R895 with a SOEing -PCR product generated from three overlapping PCR products made of the 5'-dnaE sequence (primers OVK251 and OVK252 using genomic DNA of R895 as template), the *lac*I and P_{lac} promoter sequences up to the ATG initiation codon (primers OVK136 and OVK237 using genomic DNA of R3311 as template) and the *dna*E sequence (primers OVK256 and OVK254 using genomic DNA of R895 as template).

Of note, an extra sequence of 7 nucleotides was found inserted between the Shine-Dalgarno and the ATG of the P_{lac} promoter in this synthetic $^{e}P_{lac}$::*dnaE* construct.

These Spc^R strains have been selected on solid THY medium supplemented by IPTG (10 µM). Next, clones were screened for their IPTG dependency for growth, and the genomic region encompassing the fused PCR fragments was fully sequenced.

ori/ter ratio measurement.

Genomic DNAs were prepared using DNeasy DNA extraction kit (Qiagen). Quantitative realtime PCR were performed on a Realplex thermocycler device using Sybergreen dye (iQ SYBR Green Supermix, Biorad) to amplify specific origin or terminus sequences. Oligonucleotides used for PCR amplification were chosen by using the 'eprimer3' program (http://bioweb.pasteur.fr). The amplified origin sequence corresponds to a 129 bps long PCR product obtained by using primers OVK36 and OVK37 that target the 7 594 - 7 722 region of the *S. pneumoniae* chromosome (Fig. S2). The terminus sequence is a 125 bps long PCR product obtained by using primers OVK46 and OVK47 that amplify the 1 046 843 – 1 046 967 region of the *S. pneumoniae* chromosome (Fig. S2). These two pairs of primers, defined as *ori* and *ter* respectively, exhibited 98% of amplification efficiency. *ori/ter* ratio were measured at different time points of the cell growth as indicated in the text and/or in the legend of the figures. Each *ori/ter* measurement has been performed from at least two independent cell cultures.

Table S1: *Streptococcus pneumoniae* strains and primers.

**Strains**	**Genotype/description**	**Source/reference**
R800	R6 derivative	
R209	R800 but *recA*::*cm*; Cm^R	(Martin et al., 1985)
DHP14	R800 but *rexB*::*spc* ; Spc^R	(Halpern et al., 2004)
R825	R800 but *comC*::*luc* (pR414), *comC*+; Ery^R	(Bergé et al., 2002)
R895	R800 but ssbB:: *luc* (pR424), *ssbB*$^+$; Cm^R	(Chastanet et al., 2001)
R2372	*comC0*; ssbB:: *luc* (pR424), *recO*::*spc*^{13C} ; Cm^R, Spc^R	(Johnston et al., 2015)
R2373	*comC0*; ssbB:: *luc* (pR424), *recR*::*Kan*^{15C}	(Johnston et al., 2015)
R2411	R895 but *recO*::*spc*^{13C}; Cm^R, Spc^R	This study
R2412	R895 but *recR*::*kan*^{15C}; Cm^R, Kan^R	This study
R3309	R825 but *recA*::*cm*; Ery^R, Cm^R	This study
R3310	R1501 but CEP-P_{lac}::*luc;* Cm^R, Kan^R	(Johnston et al., 2015)
R3311	R3310 but *aad9;* Kan^R, Spc^R	This study
R3315	R895 but $^eP_{lac}$::*dnaE*; Cm^R; Spc^R	This study
R3316	R895 but $^eP_{lac}$::*dnaC;* Cm^R, Spc^R	This study
R3317	R895 but $^eP_{lac}$::*dnaA;* Cm^R, Spc^R	This study
R3318	R895 but CEP-P_{lac}::*dnaA;* Cm^R, Kan^R	This study
R3319	R895 but CEP-P_{lac}::*yabA;* Cm^R, kan^R	This study
R3320	R2780 but $^eP_{lac}$::*dnaA;* Cm^R, Spc^R	This study
R4036	*comC*::pR414 (*luc*); *rexB*::*spc*	This study

Primers	Sequence*; gene; position§	
OVK28	GACACATAGATGGCGTCGCTAGTA; upstream *aad9*; -286	
OVK134	ATTCTCAGGCGGTCAACGTCAACG; internal to *amiF*; +450	
OVK135	**TTCGTCCTTTCTTTTTTTGTCGAC**AAAAAGGCCATCCGTCAGGATGGC; downstream to *amiF*; +1225	
OVK136	GTCGACAAAAAAAGAAAGGACGAA; downstream to *lacI;* +1095	
OVK169	ATGTACACCTCCTTAAGCTTAATTGTT, upstream to *lacI*; -201	
OVK139	TGGAATTGACTCGATAGCTTTAAC, internal to *treP;* +127	
OVK170	**AACAATTAAGCTTAAGGAGGTGTACAT**ATGCATATGAAGAATATTAAGAAA; internal to *I-sceI*; +1	
OVK208	**ACTCCGCTATCGCTACGGGGATC**TTATTTTAAAAAGGTTTCACTAGAGA; internal to *I-sceI*; +689	
OVK172	GATCCCCGTAGCGATAGCGGAGT, upstream to *kan*; -95	
OVK181	**AGTTACGCTAGGGATAACAGGGTAATATAG**TGCGCGTCGAAGGTTATACCAAAAAA; downstream to *kdga; +1454*	
OVK301	GGC<u>CTGCAG</u>ATTCTCAGGCGGTCAACGTCAACG; internal to *amiF*; +450	
OVK304	GGCC<u>GAATTC</u>TGGAATTGACTCGATAGCTTTAAC; internal to *treP;* +127	
OVK230	**CACTCCGCTATCGCTACGGGGATC**CTACTCCCTGTATAGCAACTCGTC; internal to *yabA*; +295	
OVK213	**CGAATTTTGACAAAAAAATGTGTTTTTCTTTGTTA** GTTTGATTTTTAATGGATAATGTG; upstream to *lacI*; -45	
OVK210	TAACAAAGAAAAACACATTTTTTTGTCAAAATTCGTTT; downstream to *aad9; +747*	
OVK214	**TACTAGCGACGCCATCTATGTGTC**CACGTGACGCGTCCCGGGGCGGCC; upstream to P_{lac}; -137	
OVK215	ATACTGTTGAGCAATTCACGTTCA; internal to *luc*; +330	
OVK196	AATGGCAAACTATCACAAGCCCATG; upstream to *dnaA; -495*	
OVK219	T**AAGCTTAAGGAGGTGTACATATGTTG**AAAGAAAAACAATTTTGGAATCG; internal to *dnaA; +1*	
OVK199	ATTTAACACGCGCATTAGGAATATTT; internal to *dnaA; +522*	

OVK251	ATCAGCTGCTAGGATAGAAAGTGG; upstream *dnaE*;-506	
OVK252	**AATTTCGTCCTTTCTTTTTTTGTCG**ATTTACACTTCTCCTCTATCCTTCTCA; upstream *dnaE*;-1	
OVK237	CACCTCCTTAAGCTTAATTGTTATC; P*lac*	
OVK256	**GATAACAATTAAGCTTAAGGAGGTGTAAGCTTACAT**TTGATCGCACAACTAGATACAAAAA; internal to *dnaE*; +1	
OVK254	CCTGCTTTCAAAAGCGTTGACCCG; internal to *dnaE*; +463	
OVK245	GGAGAAAGAAGAATGAAAGTAATC; upstream *dnaC*; -485	
OVK246	**AATTTCGTCCTTTCTTTTTTTGTCGAC**AGACTTCCCTTCCTTTTACAATC;upstream *dnaC*; -1	
OVK247	**GATAACAATTAAGCTTAAGGAGGTGTACAT**ATGGCAGAAGTAGAAGAGTTACGA; internal to *dnaC*; +1	
OVK248	CACATCTCGAATGTTCTTAAACCC; internal to *dnaC; +968*	
OVK3	GGCCGGATCCTTATTTGATTTTCTTTTTGATTGA; internal to *dnaA*; +1339	
OVK36	GGATTTACAGAATTGGGCTCTG;*ori*	
OVK37	CTGCGAATACAATTCAAAACCA; *ori*	
OVK46	CGATTTTACCGGTATTTTGCAT;*ter*	
OVK47	TGGATCTATGCATATCGCTGAC;*ter*	

Cm, chloramphenicol; Ery, erythromycin; Kan, kanamycin; spc, spectinomycin; * Sequences shown in bold in OVK135 (and OVK218, OVK252, OVK246), OVK 141(and OVK229, OVK170, OVK219), OVK230 (and OVK208), OVK214, OVK213 and OVK256 (and OVK247) are complementary to OVK136, OVK169, OVK172, OVK28, OVK210 and OVK237, respectively. Positive or negative numbers are given respect to the ATG of the corresponding gene to localize the primer position. Sequences underlined in OVK179 and OVK301correspond to a PstI site, to a EcoRI site in OVK304 and a BamH1 site in OVK3.

Acknowledgements

We particularly thank Dave Lane for critical reading and helpful and editing of the manuscript, as well as Mathieu Bergé, Calum Johnston, Nathalie Campo for their critical reading of the manuscript. This work was funded by the Agence Nationale de la Recherche (Grants ANR-10-BLAN-1331 and ANR-13-BSV8-0022).

Author contributions :

V. Khemicci and M. Prudhomme, Conceptualization, Data curation, Formal analysis, Investigation, Methodology, Writing - original draft, P. Polard, Conceptualization, Investigation, Writing – original draft, review and editing, Supervision, Funding acquisition.

References

Bergé, M., Moscoso, M., Prudhomme, M., Martin, B., and Claverys, J.-P. (2002). Uptake of transforming DNA in Gram-positive bacteria: a view from Streptococcus pneumoniae. Mol. Microbiol. *45*, 411–421.

Bergé, M.J., Mercy, C., Mortier-Barrière, I., VanNieuwenhze, M.S., Brun, Y.V., Grangeasse, C., Polard, P., and Campo, N. (2017). A programmed cell division delay preserves genome integrity during natural genetic transformation in Streptococcus pneumoniae. Nat. Commun. *8*, 1621.

Brown, N.C. (1970). 6-(p-Hydroxyphenylazo)-uracil: A Selective Inhibitor of Host DNA Replication in Phage-Infected Bacillus subtilis. Proc. Natl. Acad. Sci. *67*, 1454–1461.

Charpentier, X., Polard, P., and Claverys, J.-P. (2012). Induction of competence for genetic transformation by antibiotics: convergent evolution of stress responses in distant bacterial species lacking SOS? Curr. Opin. Microbiol. *15*, 570–576.

Chastanet, A., Prudhomme, M., Claverys, J.P., and Msadek, T. (2001). Regulation of Streptococcus pneumoniae clp genes and their role in competence development and stress survival. J. Bacteriol. *183*, 7295–7307.

Chen, J.D., and Morrison, D.A. (1987). Modulation of competence for genetic transformation in Streptococcus pneumoniae. J. Gen. Microbiol. *133*, 1959–1967.

Claverys, J.-P., Prudhomme, M., and Martin, B. (2006). Induction of competence

regulons as a general response to stress in gram-positive bacteria. Annu. Rev. Microbiol. *60*, 451–475.

Cox, M.M. (2007). Regulation of bacterial RecA protein function. Crit. Rev. Biochem. Mol. Biol. *42*, 41–63.

Dubnau, D., and Blokesch, M. (2019). Mechanisms of DNA Uptake by Naturally Competent Bacteria. Annu. Rev. Genet. *53*, 217–237.

Engelmoer, D.J.P., and Rozen, D.E. (2011). Competence increases survival during stress in Streptococcus pneumoniae. Evol. Int. J. Org. Evol. *65*, 3475–3485.

Erill, I., Campoy, S., and Barbé, J. (2007). Aeons of distress: an evolutionary perspective on the bacterial SOS response. FEMS Microbiol. Rev. *31*, 637–656.

Goranov, A.I., Breier, A.M., Merrikh, H., and Grossman, A.D. (2009). YabA of Bacillus subtilis controls DnaA-mediated replication initiation but not the transcriptional response to replication stress. Mol. Microbiol. *74*, 454–466.

Halpern, D., Gruss, A., Claverys, J.-P., and El-Karoui, M. (2004). rexAB mutants in Streptococcus pneumoniae. Microbiol. Read. Engl. *150*, 2409–2414.

Johnston, C., Caymaris, S., Zomer, A., Bootsma, H.J., Prudhomme, M., Granadel, C., Hermans, P.W.M., Polard, P., Martin, B., and Claverys, J.-P. (2013). Natural genetic transformation generates a population of merodiploids in Streptococcus pneumoniae. PLoS Genet. *9*, e1003819.

Johnston, C., Campo, N., Bergé, M.J., Polard, P., and Claverys, J.-P. (2014a). Streptococcus pneumoniae, le transformiste. Trends Microbiol. *22*, 113–119.

Johnston, C., Martin, B., Fichant, G., Polard, P., and Claverys, J.-P. (2014b). Bacterial transformation: distribution, shared mechanisms and divergent control. Nat. Rev. Microbiol. *12*, 181–196.

Johnston, C., Mortier-Barrière, I., Granadel, C., Polard, P., Martin, B., and Claverys, J.-P. (2015). RecFOR is not required for pneumococcal transformation but together with XerS for resolution of chromosome dimers frequently formed in the process. PLoS Genet. *11*, e1004934.

Johnston, C., Hauser, C., Hermans, P.W.M., Martin, B., Polard, P., Bootsma, H.J., and Claverys, J.-P. (2016). Fine-tuning of choline metabolism is important for pneumococcal colonization. Mol. Microbiol. *100*, 972–988.

Kowalczykowski, S.C. (2015). An Overview of the Molecular Mechanisms of Recombinational DNA Repair. Cold Spring Harb. Perspect. Biol. *7*.

Martin, B., Prats, H., and Claverys, J.P. (1985). Cloning of the hexA mismatch-repair gene of Streptococcus pneumoniae and identification of the product. Gene *34*, 293–303.

Martin, B., Granadel, C., Campo, N., Hénard, V., Prudhomme, M., and Claverys, J.-P. (2010). Expression and maintenance of ComD-ComE, the two-component signal-transduction system that controls competence of Streptococcus pneumoniae. Mol. Microbiol. *75*, 1513–1528.

Martin, B., Soulet, A.-L., Mirouze, N., Prudhomme, M., Mortier-Barrière, I., Granadel, C., Noirot-Gros, M.-F., Noirot, P., Polard, P., and Claverys, J.-P. (2013). ComE/ComE~P interplay dictates activation or extinction status of pneumococcal X-state (competence). Mol. Microbiol. *87*, 394–411.

Merrikh, H., and Grossman, A.D. (2011). Control of the replication initiator DnaA by an anti-cooperativity factor. Mol. Microbiol. *82*, 434–446.

Michel, B., and Leach, D. (2012). Homologous Recombination-Enzymes and Pathways. EcoSal Plus *5*.

Michel, B., Sinha, A.K., and Leach, D.R.F. (2018). Replication Fork Breakage and Restart in Escherichia coli. Microbiol. Mol. Biol. Rev. MMBR *82*.

Mirouze, N., Bergé, M.A., Soulet, A.-L., Mortier-Barrière, I., Quentin, Y., Fichant, G., Granadel, C., Noirot-Gros, M.-F., Noirot, P., Polard, P., et al. (2013). Direct involvement of DprA, the transformation-dedicated RecA loader, in the shut-off of pneumococcal competence. Proc. Natl. Acad. Sci. U. S. A. *110*, E1035-1044.

Mortier-Barrière, I., Velten, M., Dupaigne, P., Mirouze, N., Piétrement, O., McGovern, S., Fichant, G., Martin, B., Noirot, P., Le Cam, E., et al. (2007). A key presynaptic role in transformation for a widespread bacterial protein: DprA conveys

incoming ssDNA to RecA. Cell *130*, 824–836.

Noirot-Gros, M.-F., Velten, M., Yoshimura, M., McGovern, S., Morimoto, T., Ehrlich, S.D., Ogasawara, N., Polard, P., and Noirot, P. (2006). Functional dissection of YabA, a negative regulator of DNA replication initiation in Bacillus subtilis. Proc. Natl. Acad. Sci. U. S. A. *103*, 2368–2373.

Ogura, Y., Imai, Y., Ogasawara, N., and Moriya, S. (2001). Autoregulation of the dnaA-dnaN operon and effects of DnaA protein levels on replication initiation in Bacillus subtilis. J. Bacteriol. *183*, 3833–3841.

Prudhomme, M., Attaiech, L., Sanchez, G., Martin, B., and Claverys, J.-P. (2006). Antibiotic stress induces genetic transformability in the human pathogen Streptococcus pneumoniae. Science *313*, 89–92.

Prudhomme, M., Berge, M., Martin, B., and Polard, P. (2016). Pneumococcal Competence Coordination Relies on a Cell-Contact Sensing Mechanism. PLoS Genet. *12*, e1006113.

Radman, M. (1975). SOS repair hypothesis: phenomenology of an inducible DNA repair which is accompanied by mutagenesis. Basic Life Sci. *5A*, 355–367.

Sanders, G.M., Dallmann, H.G., and McHenry, C.S. (2010). Reconstitution of the B. subtilis replisome with 13 proteins including two distinct replicases. Mol. Cell *37*, 273–281.

Slager, J., Kjos, M., Attaiech, L., and Veening, J.-W. (2014a). Antibiotic-induced replication stress triggers bacterial competence by increasing gene dosage near the origin. Cell *157*, 395–406.

Slager, J., Kjos, M., Attaiech, L., and Veening, J.-W. (2014b). Antibiotic-induced replication stress triggers bacterial competence by increasing gene dosage near the origin. Cell *157*, 395–406.

Soufo, C.D., Soufo, H.J.D., Noirot-Gros, M.-F., Steindorf, A., Noirot, P., and Graumann, P.L. (2008). Cell-cycle-dependent spatial sequestration of the DnaA replication initiator protein in Bacillus subtilis. Dev. Cell *15*, 935–941.

Weyder, M., Prudhomme, M., Bergé, M., Polard, P., and Fichant, G. (2018).

Dynamic Modeling of Streptococcus pneumoniae Competence Provides Regulatory Mechanistic Insights Into Its Tight Temporal Regulation. Front. Microbiol. *9*, 1637.

Witkin, E.M. (1976). Ultraviolet mutagenesis and inducible DNA repair in Escherichia coli. Bacteriol. Rev. *40*, 869–907.

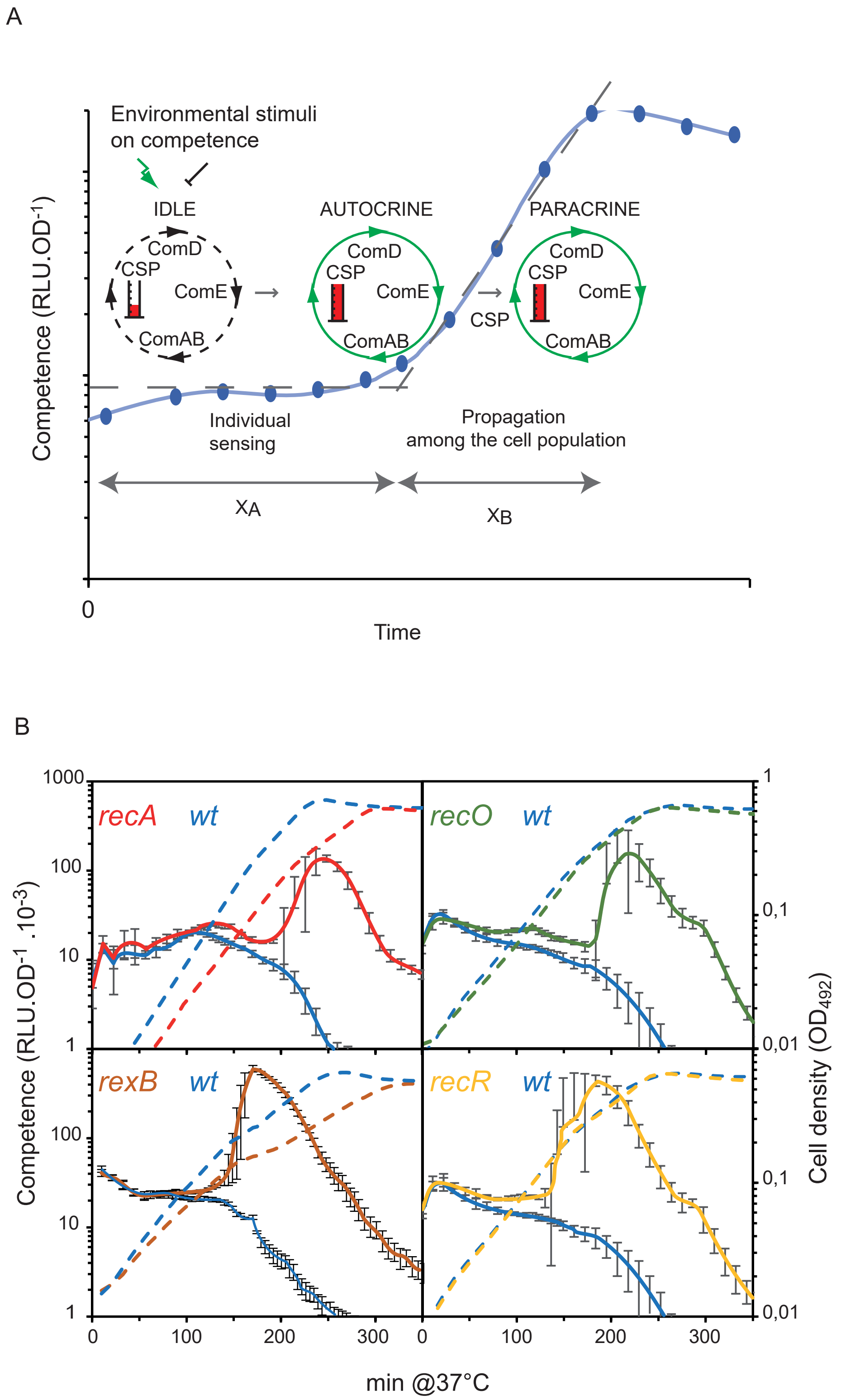
A
Environmental stimuli
on competence
IDLE
AUTOCRINE
PARACRINE
ComD
CSP
ComE
ComAB
CSP
Individual
sensing
Propagation
among the cell population
XA
XB
Competence (RLU.OD-1)
0
Time
B
recA
wt
recO
rexB
recR
1000
100
10
1
0,1
0,01
Competence (RLU.OD-1 .10-3)
Cell density (OD492)
0
100
200
300
min @37°C

Figure 1

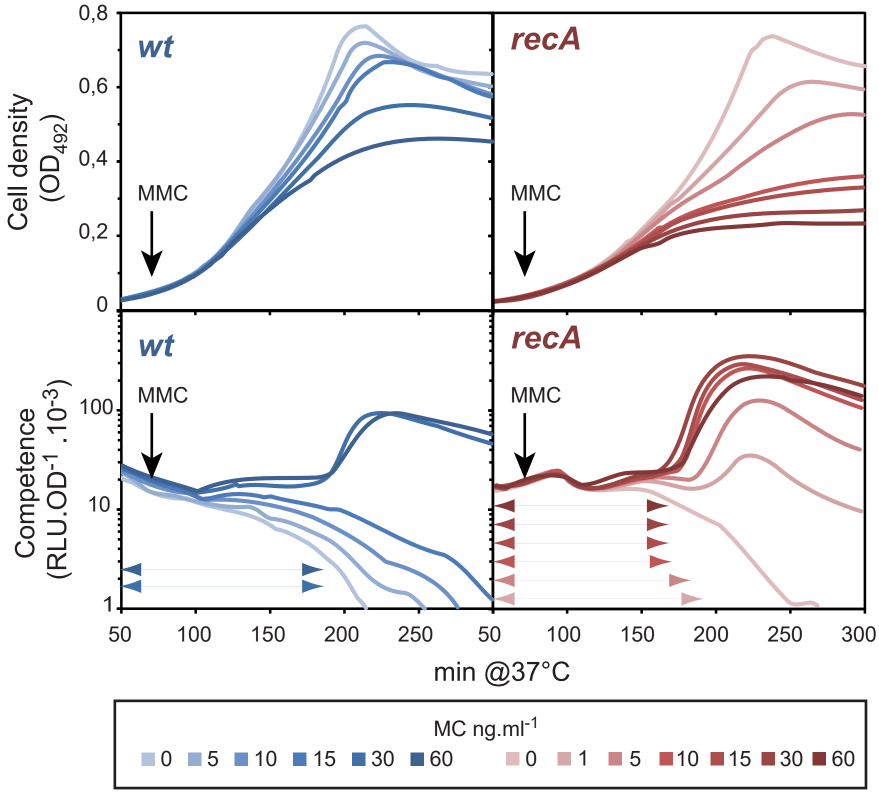

wt
recA
MMC
Cell density
(OD_{492})
0,8
0,6
0,4
0,2
0
Competence
(RLU.OD^{-1} .10^{-3})
100
10
1
50
100
150
200
250
300
min @37°C
MC ng.ml^{-1}
0 5 10 15 30 60
0 1 5 10 15 30 60

Figure 2

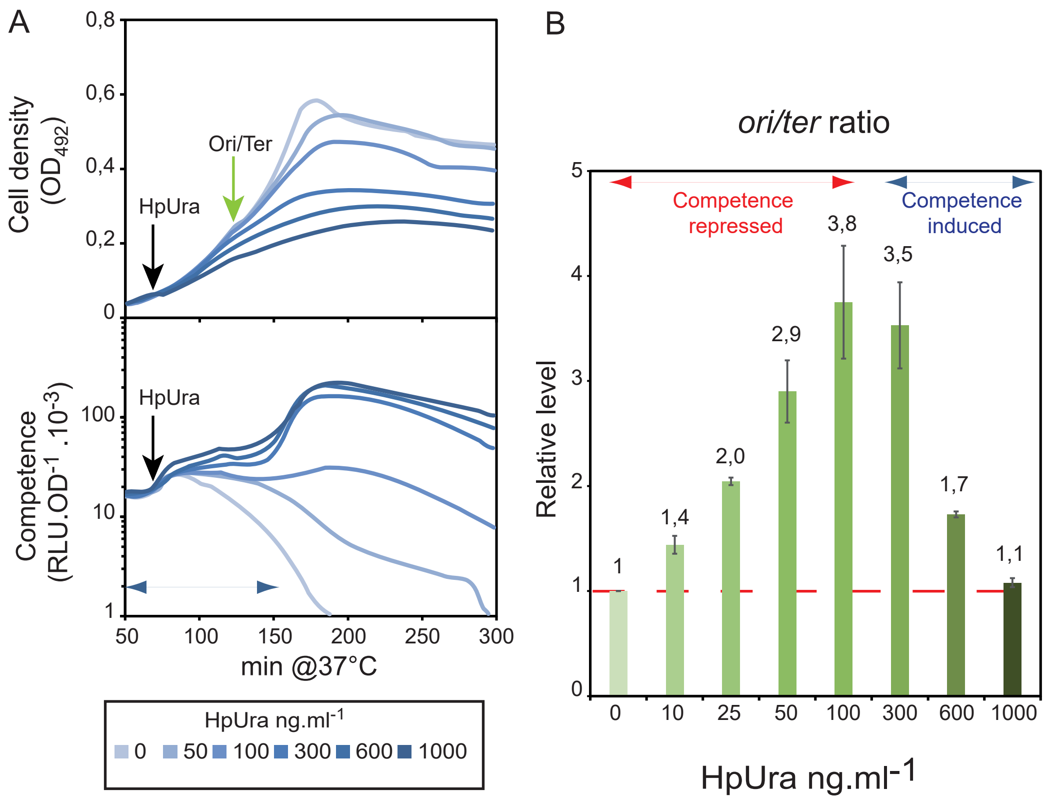
A
0,8
0,6
0,4
0,2
0
Cell density
(OD492)
Ori/Ter
HpUra
HpUra
100
10
1
Competence
(RLU.OD-1 .10-3)
50
100
150
200
250
300
min @37°C
HpUra ng.ml-1
0
50
100
300
600
1000
B
ori/ter ratio
5
4
3
2
1
0
Relative level
Competence
repressed
Competence
induced
1
1,4
2,0
2,9
3,8
3,5
1,7
1,1
0
10
25
50
100
300
600
1000
HpUra ng.ml-1

Figure 3

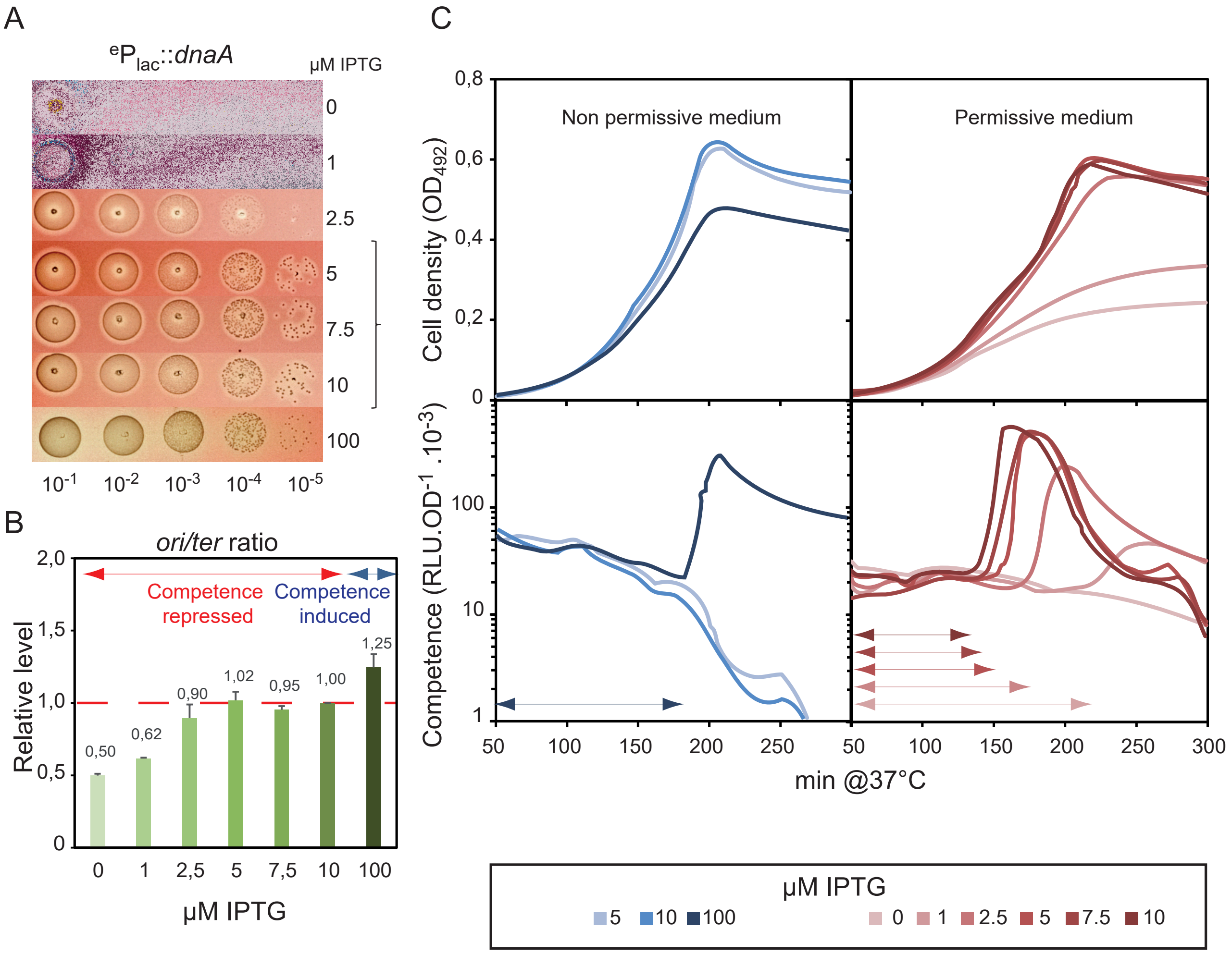
A
ePlac::dnaA
µM IPTG
0
1
2.5
5
7.5
10
100
10-1 10-2 10-3 10-4 10-5
B
ori/ter ratio
Competence repressed
Competence induced
Relative level
0,50 0,62 0,90 1,02 0,95 1,00 1,25
0 1 2,5 5 7,5 10 100
µM IPTG
C
Non permissive medium
Permissive medium
Cell density (OD492)
Competence (RLU.OD-1 .10-3)
min @37°C
µM IPTG
5 10 100
0 1 2.5 5 7.5 10

Figure 4

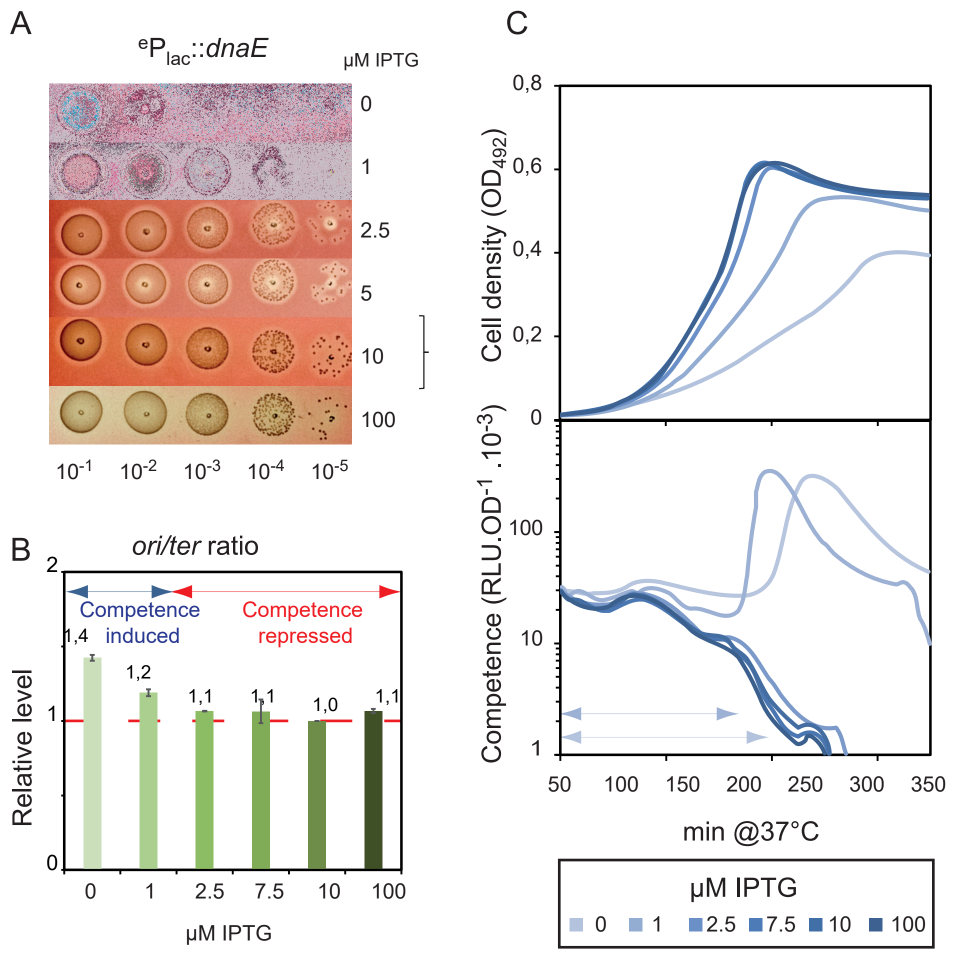
A
ePlac::dnaE
µM IPTG
0
1
2.5
5
10
100
10-1 10-2 10-3 10-4 10-5
B
ori/ter ratio
Competence induced
Competence repressed
Relative level
1,4
1,2
1,1
1,1
1,0
1,1
0 1 2.5 7.5 10 100
µM IPTG
C
Cell density (OD492)
0,8
0,6
0,4
0,2
0
Competence (RLU.OD-1 .10-3)
100
10
1
50 100 150 200 250 300 350
min @37°C
µM IPTG
0 1 2.5 7.5 10 100

Figure 5

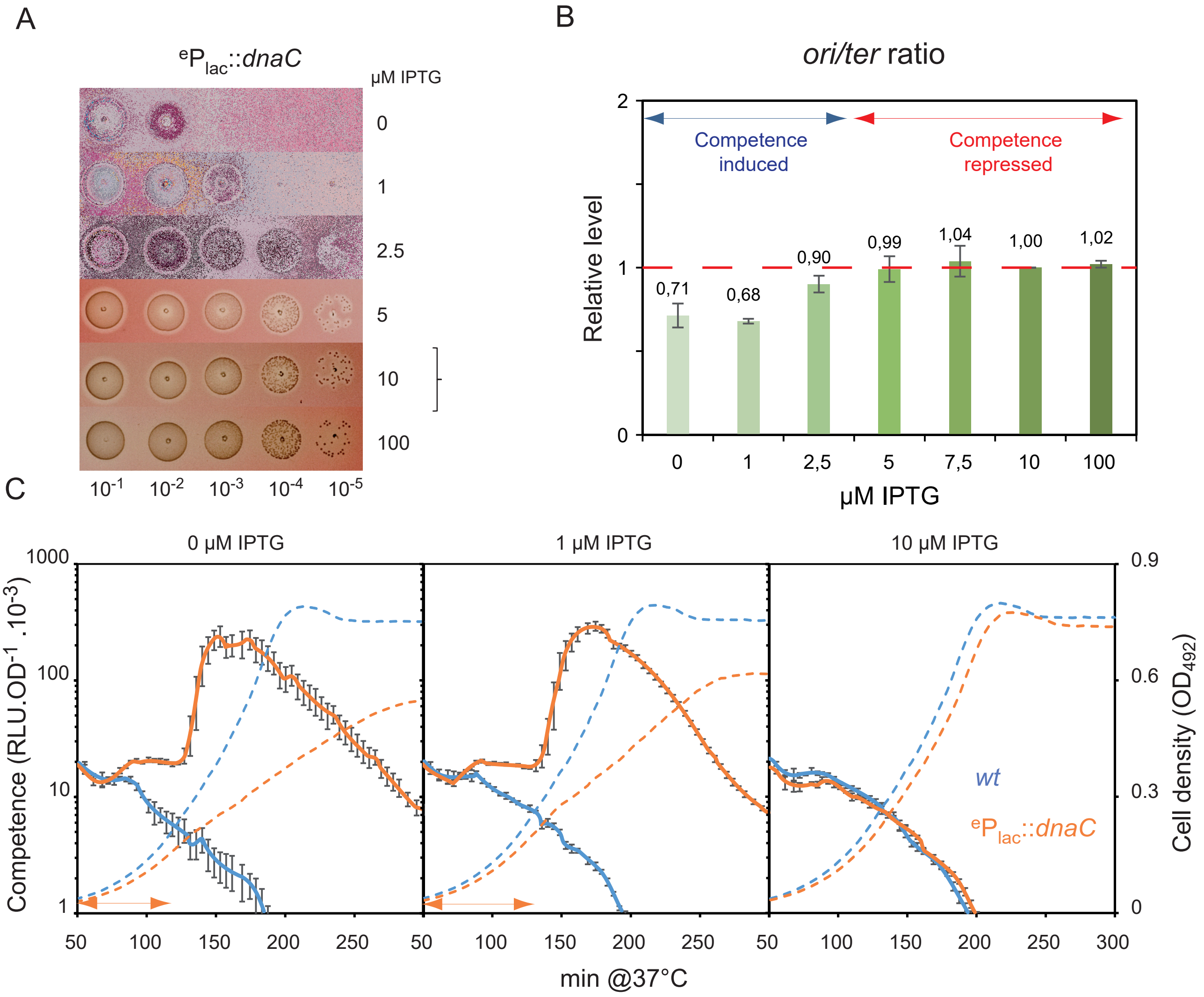
A
ePlac::dnaC
µM IPTG
0
1
2.5
5
10
100
10-1 10-2 10-3 10-4 10-5
B
ori/ter ratio
Competence induced
Competence repressed
Relative level
0,71
0,68
0,90
0,99
1,04
1,00
1,02
0 1 2,5 5 7,5 10 100
µM IPTG
C
0 µM IPTG
1 µM IPTG
10 µM IPTG
Competence (RLU.OD-1 .10-3)
Cell density (OD492)
wt
ePlac::dnaC
min @37°C

Figure 6

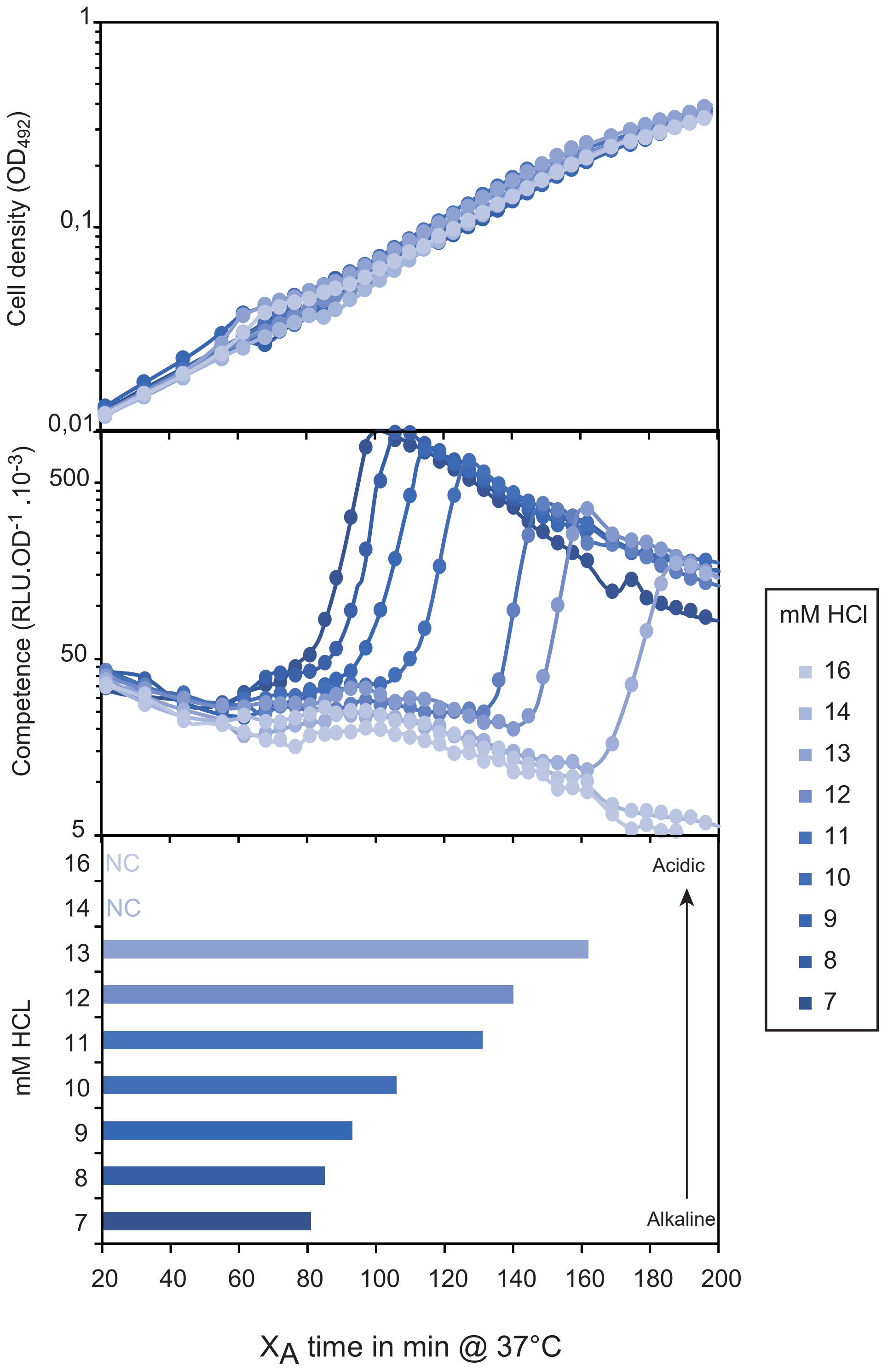

Cell density (OD_{492})
1
0,1
0,01
Competence (RLU.OD^{-1} . 10^{-3})
500
50
5
mM HCL
16
14
13
12
11
10
9
8
7
NC
NC
Acidic
Alkaline
20
40
60
80
100
120
140
160
180
200
X_A time in min @ 37°C
mM HCl
16
14
13
12
11
10
9
8
7

Figure S1

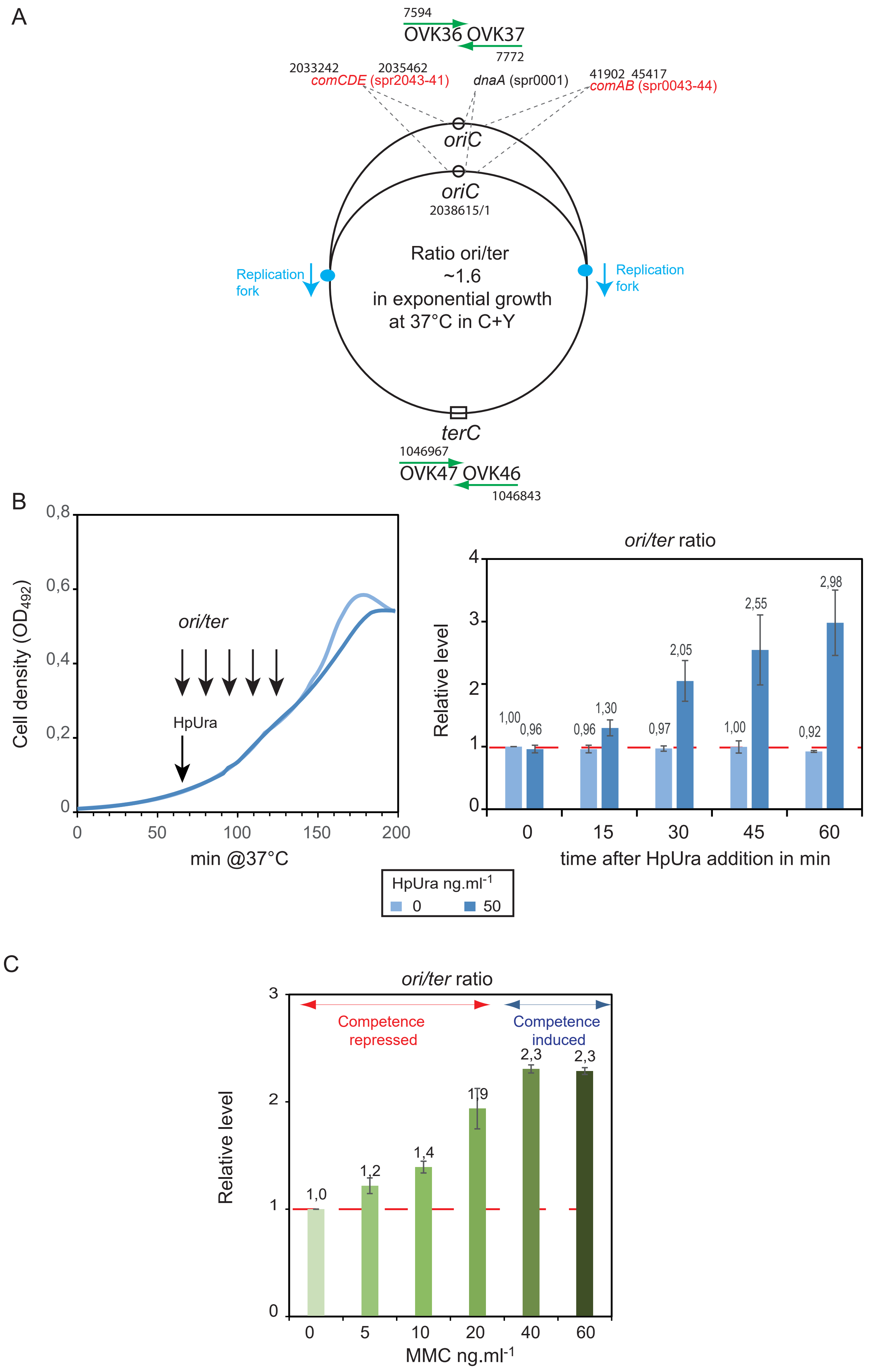
A
7594
OVK36 OVK37
7772
2033242 2035462
comCDE (spr2043-41)
dnaA (spr0001)
41902 45417
comAB (spr0043-44)
oriC
oriC
2038615/1
Ratio ori/ter
~1.6
in exponential growth
at 37°C in C+Y
Replication fork
Replication fork
terC
1046967
OVK47 OVK46
1046843
B
Cell density (OD492)
0,8
0,6
0,4
0,2
0
ori/ter
HpUra
0 50 100 150 200
min @37°C
ori/ter ratio
Relative level
4
3
2
1
0
1,00 0,96
0,96 1,30
0,97 2,05
1,00 2,55
0,92 2,98
0 15 30 45 60
time after HpUra addition in min
HpUra ng.ml-1
0 50
C
ori/ter ratio
Relative level
3
2
1
0
Competence repressed
Competence induced
1,0
1,2
1,4
1,9
2,3
2,3
0 5 10 20 40 60
MMC ng.ml-1

Figure S2

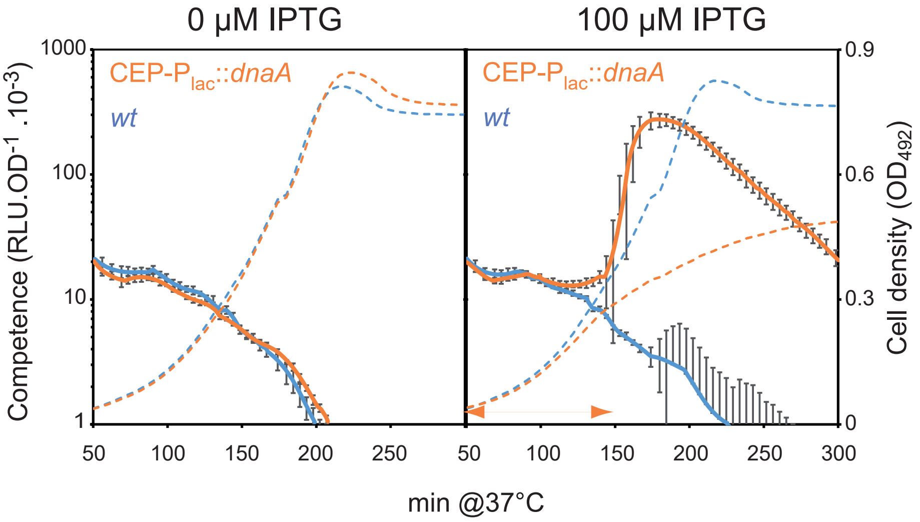

0 µM IPTG
100 µM IPTG
CEP-Plac::dnaA
wt
CEP-Plac::dnaA
wt
1000
100
10
1
Competence (RLU.OD-1 .10-3)
0.9
0.6
0.3
0
Cell density (OD492)
50
100
150
200
250
50
100
150
200
250
300
min @37°C

Figure S3

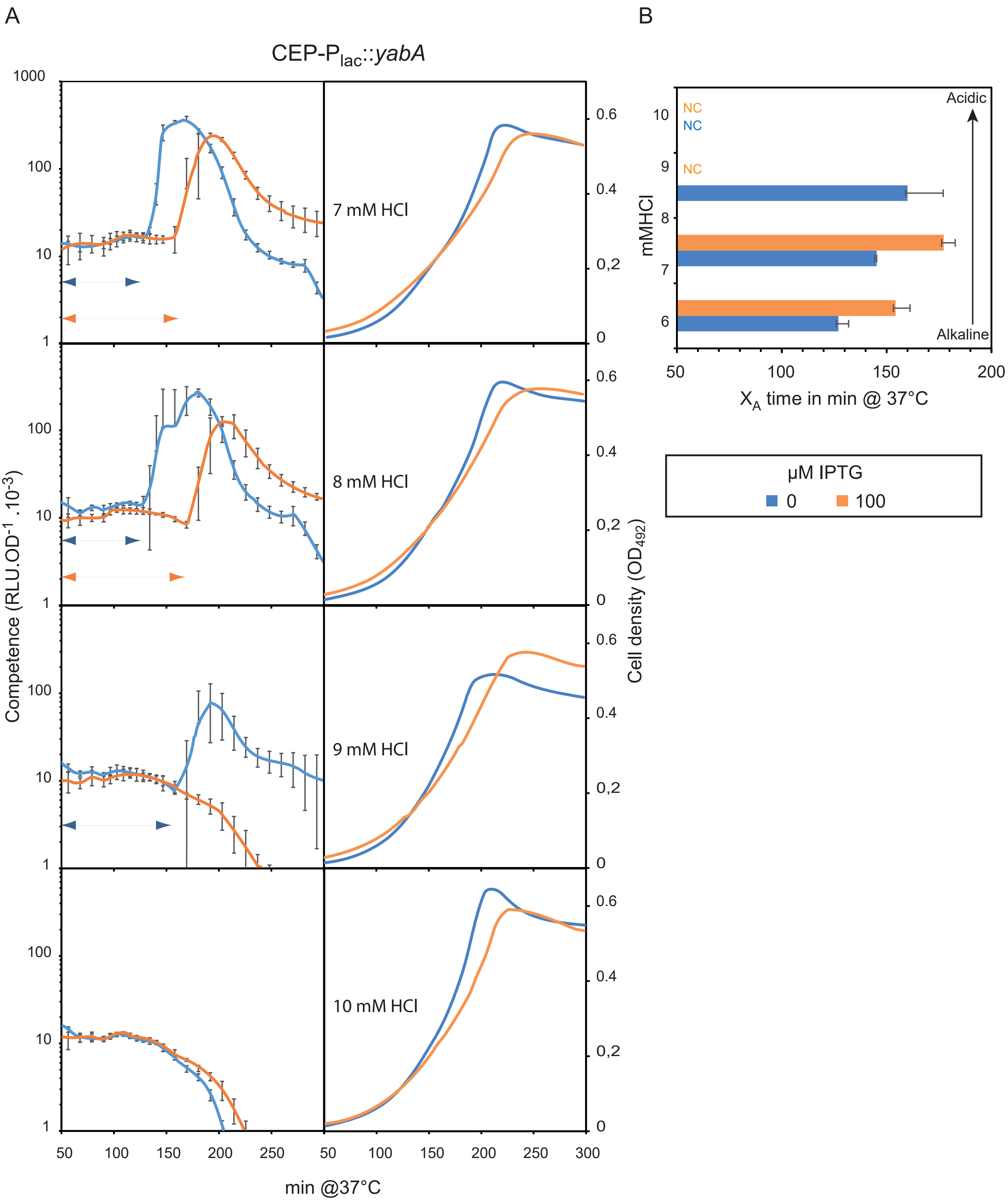
A
B
CEP-P lac::yabA
7 mM HCl
8 mM HCl
9 mM HCl
10 mM HCl
Competence (RLU.OD-1 .10-3)
Cell density (OD492)
min @37°C
mMHCl
NC
Acidic
Alkaline
XA time in min @ 37°C
µM IPTG
0
100

Figure S4

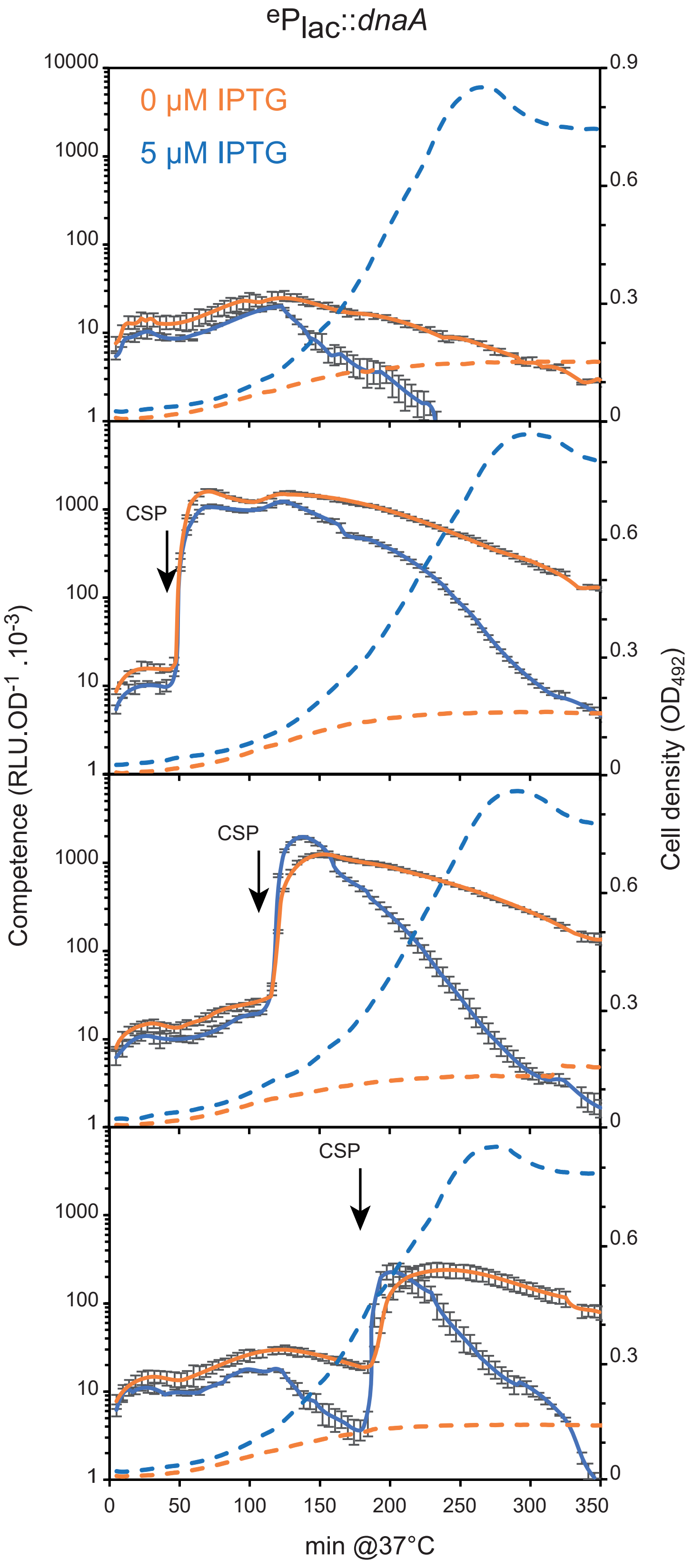

ePlac::dnaA
0 µM IPTG
5 µM IPTG
CSP
CSP
CSP
Competence (RLU.OD-1 .10-3)
Cell density (OD492)
min @37°C
10000
1000
100
10
1
0.9
0.6
0.3
0
0
50
100
150
200
250
300
350

Figure S5
